# Bortezomib enhances cancer cell death by blocking the autophagic flux through stimulating ERK phosphorylation

**DOI:** 10.1038/cddis.2014.468

**Published:** 2014-11-06

**Authors:** C Kao, A Chao, C-L Tsai, W-C Chuang, W-P Huang, G-C Chen, C-Y Lin, T-H Wang, H-S Wang, C-H Lai

**Affiliations:** 1 Department of Obstetrics and Gynecology, Linkou Medical Center, Chang Gung Memorial Hospital, Taoyuan, Taiwan; 2 Graduate Institute of Biomedical Sciences, College of Medicine, Chang Gung University, Taoyuan, Taiwan; 3 Department of Life Science, National Taiwan University, Taipei, Taiwan; 4 Institute of Biological Chemistry, Academia Sinica, Taipei 115, Taiwan; 5 Genomic Medicine Research Core Laboratory, Linkou Medical Center, Chang Gung Memorial Hospital, Taoyuan, Taiwan; 6 School of Traditional Chinese Medicine, College of Medicine, Chang Gung University, Taoyuan, Taiwan; 7 Graduate Institute of Clinical Medical Sciences, College of Medicine, Chang Gung University, Taoyuan, Taiwan

## Abstract

The antitumor activity of an inhibitor of 26S proteasome bortezomib (Velcade) has been observed in various malignancies, including colon cancer, prostate cancer, breast cancer, and ovarian cancer. Bortezomib has been proposed to stimulate autophagy, but scientific observations did not always support this. Interactions between ERK activity and autophagy are complex and not completely clear. Autophagy proteins have recently been shown to regulate the functions of ERK, and ERK activation has been found to induce autophagy. On the other hand, sustained activation of ERK has also been shown to inhibit the maturation step of the autophagy process. In this study, we sought to identify the mechanism of autophagy regulation in cancer cells treated with bortezomib. Our results indicate that bortezomib blocked the autophagic flux without inhibiting the fusion of the autophagosome and lysosome. In ovarian cancer, as well as endometrial cancer and hepatocellular carcinoma cells, bortezomib inhibited protein degradation in lysosomes by suppressing cathepsins, which requires the participation of ERK phosphorylation, but not JNK or p38. Our findings that ERK phosphorylation reduced cathepsins further explain how ERK phosphorylation inhibits the autophagic flux. In conclusion, bortezomib may induce ERK phosphorylation to suppress cathepsin B and inhibit the catalytic process of autophagy in ovarian cancer and other solid tumors. The inhibition of cisplatin-induced autophagy by bortezomib can enhance chemotherapy efficacy in ovarian cancer. As we also found that bortezomib blocks the autophagic flux in other cancers, the synergistic cytotoxic effect of bortezomib by abolishing chemotherapy-related autophagy may help us develop strategies of combination therapies for multiple cancers.

Ovarian cancer leads to the most deaths of all gynecologic cancers.^1^ Early diagnosis of ovarian cancer is generally difficult because of the late clinical presentation in its natural history.^2^ Surgery followed by adjuvant chemotherapy is the mainstay of the treatment. Advances in adjuvant chemotherapy such as dose-dense paclitaxel^3^ and the addition of an angiogenesis inhibitor^4, 5^ are increasing the progression-free survival and overall survival. However, even now, complete cures are still rarely accomplished, and current agents for the treatment of recurrent disease have only been modestly effective.^2^ There is an urgent need to investigate drugs with different mechanisms of action in order to develop a better therapeutic strategy in ovarian cancer.

Bortezomib (Velcade, formerly known as PS-341), an inhibitor of the 26S proteasome, is approved for the treatment of multiple myeloma.^6^ Its antitumor activity has also been observed in various malignancies, including colon cancer, prostate cancer, breast cancer, and ovarian cancer.^7, 8, 9, 10, 11^ In addition to proteasome inhibition, one of the antimyeloma mechanisms of bortezomib is the inhibition of transcription factor nuclear factor-kappa beta (NF-*κ*B).^6^ Blocking the NF-*κ*B pathway increased the sensitivity of cancer cells to chemotherapy and enhanced their cellular susceptibility to apoptosis.^6^ Recently, bortezomib has been shown to regulate autophagy in breast cancer, melanoma, head and neck cancer, hepatocellular carcinoma, and prostate cancer.^9, 12, 13, 14, 15, 16, 17^ However, bortezomib-related autophagy regulation has not been reported in ovarian cancer yet.

Autophagy is a conserved process in all eukaryotic cells and contributes to organelle turnover, protein degradation, and differentiation.^18^ Autophagy begins with the formation of autophagosomes that selectively engulf organelles and other materials in the cytoplasm. Light chain 3-II (LC3-II) is important in the elongation of double membrane during formation of the autophagosome.^19^ The mature autophagosome then fuses with the lysosome, forming the autolysosome. Subsequently, autolysosomal components are degraded by lysosomal catalytic enzymes. Morphologically, initial/immature autophagic vacuoles (Avi) are cytosolic portions enclosed by double membranes. Upon fusion with endosome, Avi become intermediate, double-membraned, autophagic vacuoles (Avi/d). Upon further fusion with lysosome, these vacuoles become double-membraned, degrading autophagic vacuoles (Avd). Complete degradation results in heterogeneous contents and single membrane structures.^20, 21, 22^ P62/sequestosome 1 (p62/SQTM1) is a useful indicator to monitor autophagy, because it decreases when autophagy is completed.^23^ P62 accumulates in autophagic-defective tumor cells.^19^ In short, p62 degradation is autophagy-dependent.^24, 25, 26^

The role of autophagy in cancer cells is complex and context-dependent.^26, 27^ During tumorigenesis, the activation of autophagy suppresses tumor development,^28^ and downregulation of autophagy has been shown to promote the aggressiveness of ovarian tumors.^29^ The autophagy marker LC3 is expressed at lower levels in ovarian cancer tissues than in benign and borderline ovarian tumors, and its expression is lower in advanced stages than in early stages of ovarian cancer.^29^ On the other hand, the upregulation of autophagy has been shown to increase the survival of clear cell ovarian cancer cells during hypoxic events.^30^ Chemotherapeutic agents induce cellular and metabolic stress that leads to pro-survival autophagy,^31^ as exemplified by increased autophagy in ovarian cancer cells treated with cisplatin.^32^ Activation of autophagy is also beneficial to ovarian cancer cells against chemotherapy, and may account for drug resistance.^33^

In this study, we sought to identify the role and mechanism of autophagy in cancer cells treated with bortezomib. Our results indicated that bortezomib blocked the autophagic flux at the autophagolysomal stage by decreasing the levels of cathepsins. More surprisingly, the suppression of cathepsins by bortezomib was mediated by the activation of ERK.

## Results

### Bortezomib specifically elicited autophagy but blocked the degradation of p62

**Figure 1:**
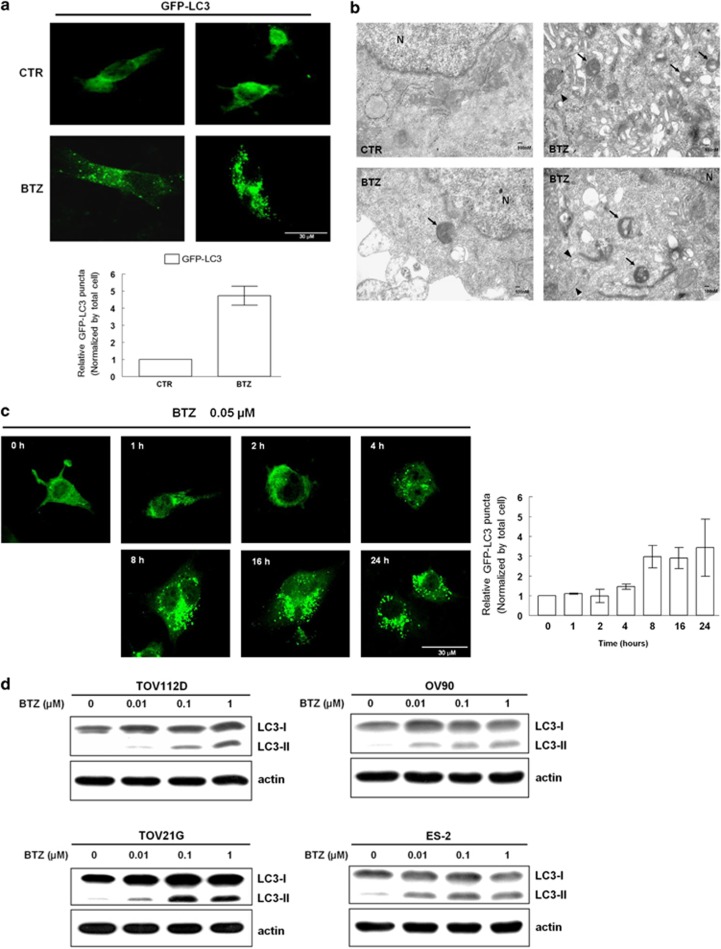
Bortezomib (BTZ) induces autophagy. (**a**) Immunofluorescent puncta formation in TOV112D cells transfected with GFP-LC3 in the presence or absence of 0.05 *μ*M BTZ for 24 h. Results shown are mean±standard error for three independent experiments. Scale bar represents 30 *μ*m. (**b**) Transmission electron microscopy showed that 24 h of BTZ treatment induced the formation of initial autophagic vacuoles (indicated with arrowhead) and degrading autophagic vacuoles (indicated with arrow). Of note, many degrading autophagic vacuoles had double membranes. Scale bar = 100 nm. (**c**) BTZ induced autophagy in a time-dependent manner. After treatment with 0.05 *μ*M BTZ for 0, 1, 2, 4, 8, 16, and 24 h, TOV112D cells were analyzed for immunofluorescent GFP-LC3 puncta formation. Results shown are mean±standard error for three independent experiments. Scale bar represents 30 *μ*m. (**d**) After treatment with designated concentrations of BTZ for 24 h, ovarian cancer cells (TOV112D, TOV21G, OV90, and ES2) were analyzed with western blots for the intensity changes in LC3-I and LC3-II, as indicated. CTR, control

Treatment with bortezomib increased GFP-LC3 puncta (Figure 1a) and the formation of a double-membraned initial autophagic vacuoles (Avi) and degrading autophagic vacuoles (Avd) (Figure 1b). Although we did not perform quantitatively morphometric analyses of Avi and Avd, we found that the majority of autophagic vacuoles were double-membraned, degrading autophagic vacuoles (Figure 1b). The observation implied that the autophagic flux was blocked at the early stage of autolysosome. The formation of GFP-LC3 puncta increased in a time-dependent manner (Figure 1c), indicating that bortezomib initiated the process of autophagy. Bortezomib also increased the levels of LC3-II in TOV112D (endometrioid type), OV90 (serous type), TOV21G and ES2 (clear cell) ovarian cancer cells in a dose-dependent manner (Figure 1d). ATG5 is involved in the elongation of double membrane in autophagosome, and beclin-1 is required for the initiation of autophagy.^19^ Increased levels of ATG5-ATG12 and beclin1 in bortezomib-treated ovarian cancer cells suggested that bortezomib induced the initial steps of autophagy (Supplementary Figure 1).

**Figure 2:**
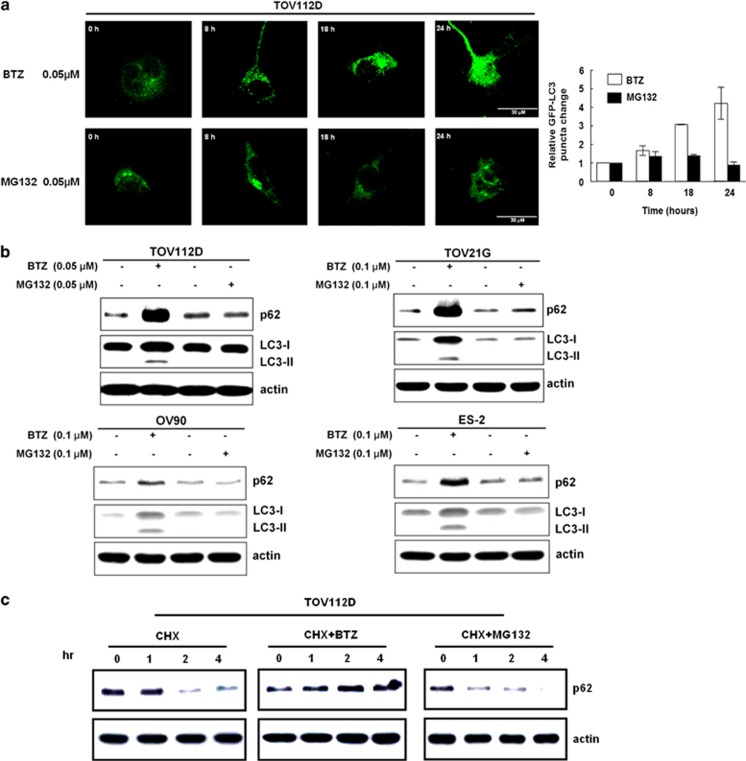
Bortezomib (BTZ) specifically initiates autophagy but blocks the degradation of p62. (**a**) Left panel: A time-dependent increase of immunofluorescent GFP-LC3 puncta formation was shown in TOV112D cells treated with 0.05 *μ*M BTZ, but not in those with 0.05 *μ*M MG132. Scale bar represents 30 *μ*m. Right panel: The numbers of puncta in every cell were counted and averaged. The fold change of puncta was normalized by the puncta number in the cells at 0 h. Results shown are mean±standard error for three independent experiments. (**b**) Ovarian cancer cell lines (TOV112D, TOV21G, OV90, and ES2) were treated with designated concentrations of BTZ or MG132 for 24 h, and the intensity changes in LC3-II and p62 were analyzed with western blots. (**c**) TOV112D cells were treated with either 0.05 *μ*M BTZ or 0.05 *μ*M MG132 in presence of 100 *μ*M cycloheximide (CHX) for 1, 2, and 4 h, and the changes in p62 levels were analyzed with western blots

Ubiquitin-proteasome system and autophagy are two important means of cellular degradation.^34^ Puncta formation of GFP-LC3 was detected at 8 h and increased throughout 24 h in bortezomib-treated cells; this was not found in the cells treated with another proteasome inhibitor MG132 at the same concentration (Figure 2a). LC3-II levels were also not increased by MG132 treatment in various types of ovarian cancer cells (Figure 2b). Of note, bortezomib, but not MG132, increased p62 levels in ovarian cancer cells (Figure 2b). P62 is normally degraded by a basal level of autophagy when new protein synthesis is blocked by the treatment with cycloheximide (Figure 2c). However, p62 levels were increased by the co-treatment with cycloheximide and bortezomib, but not by the co-treatment with cycloheximide and MG132 (Figure 2c). These results collectively indicate that the degradation of p62 was specifically inhibited by bortezomib. Although 0.1 *μ*M of MG132 did not increase autophagosome puncta and LC3-II levels, a much higher concentration (5 *μ*M) of MG132 did induce autophagosome and LC3-II levels (Supplementary Figure 2).

Bortezomib is a known inhibitor of the NF-*κ*B signaling pathway, and the activation of NF-*κ*B was shown to suppress autophagy.^35^ If this is the case, then bortezomib should activate autophagy and eliminate p62.^23^ However, bortezomib inhibited the NF-*κ*B signaling pathway in seven ovarian cancer lines by increasing I*κ*B phosphorylation, but also increased p62 levels in eight ovarian cancer cells (Supplementary Figure 3). Inhibition of proteasome by either bortezomib or MG132 was confirmed by the presence of increased levels of ubiquitinated proteins (Supplementary Figure 4). These results suggested that bortezomib suppressed p62 degradation via a NF-*κ*B-independent, proteasome-independent mechanism.

### Bortezomib blocked p62 degradation during autophagy but did not inhibit the fusion of lysosome and autophagosome

**Figure 3:**
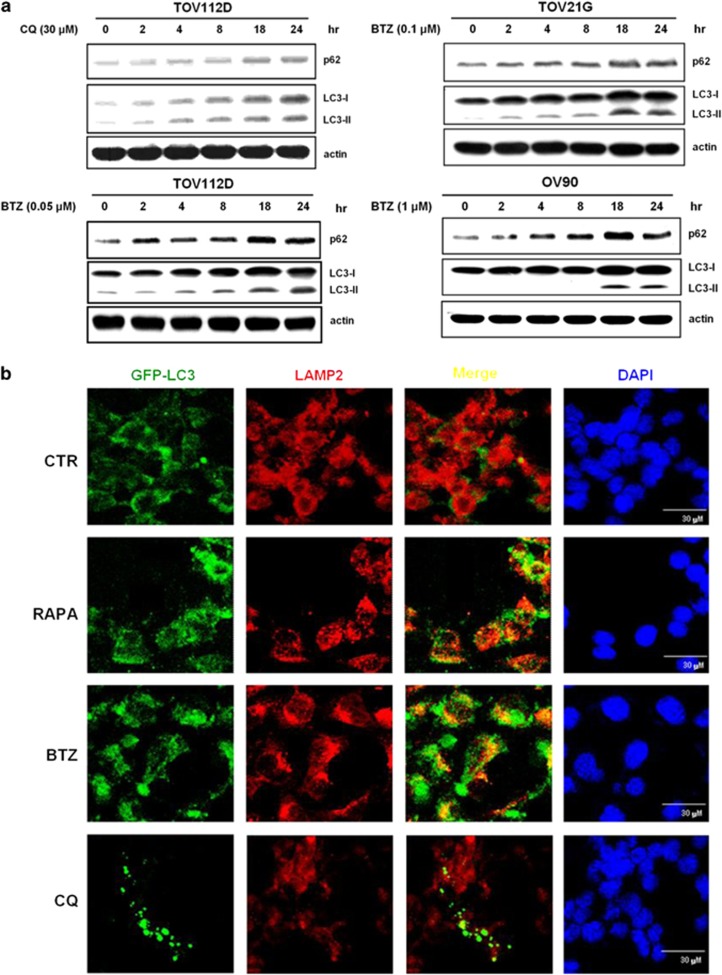
Bortezomib (BTZ) inhibits lysosome functions after the fusion of autophagosome and lysosome. (**a**) Ovarian cancer cells (TOV112D, TOV21G, and OV90) were treated with designated concentrations of BTZ for designated hours, and the level changes in LC3-I and p62 were analyzed with western blots. Treatment with 30 *μ*M chloroquine (CQ) in TOV112D cells for the same time periods was used to show the effects of fusion disruption between autophagosome and lysosome. (**b**) After being transfected with GFP-LC3 for 72 h, TOV112D cells were treated with 0.05 *μ*M BTZ, 5 *μ*M rapamycin (RAPA), or 30 *μ*M chloroquine (CQ) for 24 h, and analyzed for LAMP-2 levels with immunofluorescent microscopy. In the cells treated with rapamycin or BTZ, the merged yellow signals of GFP-LC3 (green) and LAMP-2 (red) indicated the co-localization of autophagosome (marked by GFP-LC3) and lysosome (marked by LAMP-2). Chloroquine blocked the fusion of autophagosome and lysosome, thus minimized yellow signals. Scale bar represents 30 *μ*m. CTR, control

Treatment with either chloroquine, an inhibitor that blocks the fusion of lysosome and autophagosome, or bortezomib increased p62 and LC3-II in a time-dependent manner in ovarian cancer cells (Figure 3a). The findings that bortezomib treatment increased p62 levels were also observed in endometrial cancer Ishikawa cells and hepatocellular cancer HepG2 cells (Supplementary Figure 5). To test whether bortezomib inhibited the fusion of lysosome and autophagosome, we used anti-LAMP2 antibody to identify lysosomes and LC3 puncta to indicate autophogasomes. Co-localization of LAMP2 and GFP-LC3 was shown by yellow signals that indicated the co-existence of red (LAMP2) and green (GFP-LC3) signals (Figure 3b). Fusion between autophagosome and lysosome was confirmed in bortezomib-treated cells (Figure 3b).

**Figure 4:**
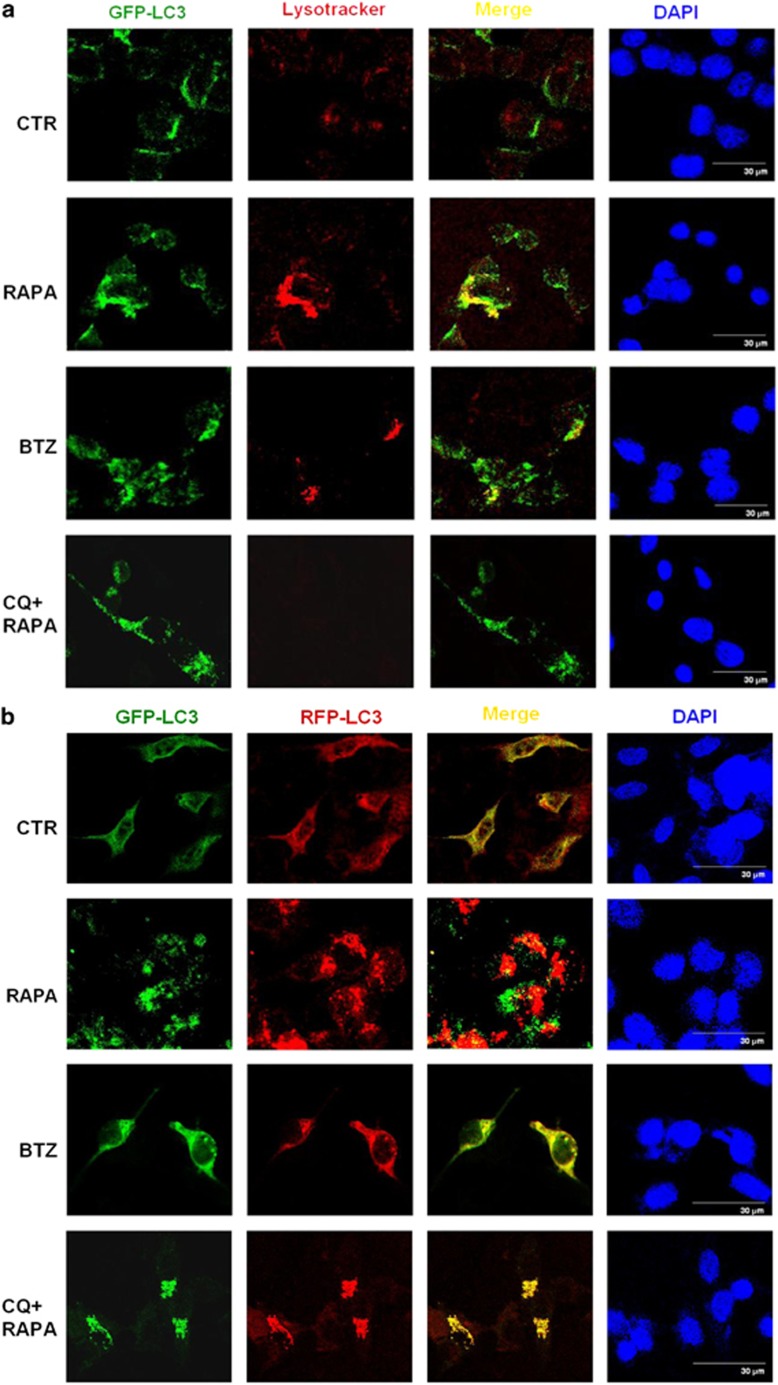
Bortezomib (BTZ) suppresses the catalytic process of autophagy in lysosome. (**a**) TOV112D cells were treated with 0.05 *μ*M BTZ, 5 *μ*M rapamycin (RAPA), or 30 *μ*M CQ for 24 h, and subjected to lysosomal analysis with 300 nM Lysotracker for 16 h. Rapamycin-treated cells were shown as the positive control of autophagy, showing the merged yellow signals of autophagosome (green fluorescence) and functional lysosome (red Lysotracker signals). Even in the cells treated with rapamycin, co-treatment with chloroquine blocked the fusion of autophagosome and lysosome and diminished lysosomal function, shown by the absence of red Lysotracker signal. (**b**) TOV112D cells were transiently transfected with GFP-RFP-LC3 vector for 72 h, and subsequently treated with 0.05 *μ*M BTZ, 5 *μ*M rapamycin (RAPA), or 30 *μ*M CQ for 24 h. Using the GFP-RFP-LC3 expression vector, autophagosomes (GFP) and autolysomes (RFP) are shown with green and red signals, respectively. Thus, the fluorescence changes can be used as an indicator for the autophagic flux. Scale bar represents 30 *μ*m. CTR, control

Lysotracker assays also revealed the co-localization of autophagosome and lysosome, where inhibition of the fusion between autophagosomes and lysosomes by chloroquine was demonstrated by the absence of the Lysotracker signal (Figure 4a). Of note, lysosomal dysfunction was shown by the decreased Lysotracker signals in bortezomib-treated cells (Figure 4a). The GFP-RFP-LC3 expression vector was used to show the flux of autophagy. Rapamycin induces the complete autophagic process, so both yellow (green GFP-LC3 and red RFP-LC3) and red (RFP only) signals were increased, indicating the presence of autophagosomes and autolysosomes, respectively (Figure 4b). Chloroquine inhibits the fusion of autophagosome and lysosome, so only autophagosomes (yellow) predominated in cells treated with both rapamycin and choloroquine (Figure 4b). Using this expression vector, the reduction of acidic lysosomal environment by bortezomib was shown by yellow puncta signal without increasing red puncta (Figure 4b). Taken together, although bortezomib itself does not inhibit the fusion of autophagosome and lysosome, it does impair lysosomal functions in the fused lysosome–autophagosome.

### Bortezomib inhibited protein degradation in lysosomes via phosphorylation of ERK, but not JNK or p38

**Figure 5:**
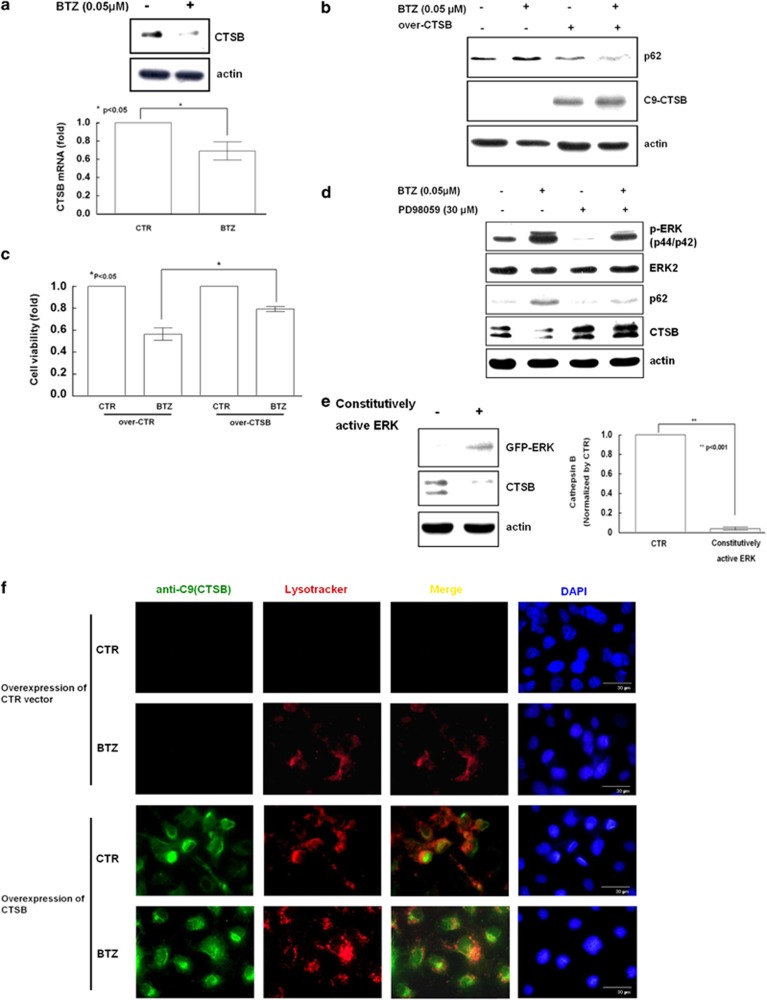
Bortezomib (BTZ) induces the phosphorylation of ERK, reduces the level of cathepsin B (CTSB), and suppresses the catalytic process in autophagic lysosomes. (**a**) TOV112D cells were treated with 0.05 *μ*M BTZ for 24 h. CTSB protein and mRNA levels were measured by immunoblotting and real-time quantitative PCR, respectively. Results shown are mean±standard error for three independent experiments. (**b**) The effects of the overexpression of CTSB for 72 h on the TOV112D cells treated with 0.05 *μ*M BTZ for 24 h were analyzed by measuring the p62 protein level and (**c**) using the MTT assays. Forced expression of C9-CTSB was detected by anti-C9 antibody with western blot analysis. Results shown are the mean±standard error for three independent experiments. (**d**) The effects induced by the treatment with 0.05 *μ*M BTZ and 30 *μ*M PD98059 for 24 h were analyzed in TOV112D cells using immunoblotting for the levels of ERK phosphorylation, CTSB, and p62. (**e**) The effect of forced expression of a constitutively active p-ERK (Y204D) for 72 h in TOV112D cells were analyzed by measuring the level of CTSB. Results shown are mean ± standard error for three independent experiments. (**f**) TOV112D cells were transfected with a C9-CTSB expression vector and treated with 0.05 *μ*M BTZ. After lysosomal labeling with 300 nM Lysotracker (red) for 16 h, the colocalization of CTSB and functional lysosomes was shown by yellow merged signals of anti-C9 (green) into Lysotracker (red). Scale bar represents 30 *μ*m

Cathepsins are important in the autophagic catalytic process.^36^ In the process to understand how bortezomib suppressed protein degradation in lysosomes, we have found that bortezomib inhibited all levels of cathepsin B/D/G (data not shown). More specifically, the treatment of TOV112D cells with bortezomib suppressed cathepsin B at both the protein and mRNA levels (Figure 5a). Forced expression of cathepsin B significantly supported p62 degradation (Figure 5b), increased signals of Lysotracker (Figure 5f), and rescued bortezomib-treated cells from cell death (Figure 5c). Concomitant with inhibiting cathepsin B and increasing p62 levels, bortezomib also stimulated ERK phosphorylation in ovarian cancer cells (Figure 5d), endometrial cancer Ishikawa cells, and hepatocellular carcinoma HepG2 cells (Supplementary Figure 5). Treatment with the ERK inhibitor-PD98059 blocked ERK phosphorylation and rescued cathepsin B levels from suppression by bortezomib, along with significant reduction of p62 (Figure 5d). Forced expression of constitutively active p-ERK (Y204D) significantly inhibited cathepsin B in TOV112D cells (Figure 5e). All of these findings were confirmed in another ovarian cancer TOV21G cells (Supplementary Figure 6). Although bortezomib treatment increased p62 levels in all of eight studied ovarian cancer cell lines, the changes in phospho-ERK and cathepsin B were not consistent in few ovarian cancer cell lines (Supplementary Figure 3), suggesting that additional regulatory mechanisms for cathepsin B exist. In addition, the proteasome inhibitor MG132 at a high concentration (5 *μ*M) also induced phospho-ERK, but it did not inhibit cathepsin B (Supplementary Figure 7). Therefore, the inhibition of cathepsin B was specific to bortezomib but not to other proteasome inhibitors.

Of note, bortezomib also stimulated JNK phosphorylation but inhibited p38 phosphorylation (Supplementary Figure 8A). However, concomitant treatment with bortezomib and the JNK inhibitor-SP600125 did not rescue cathepsin B levels (Supplementary Figure 8B). In fact, inhibition of JNK further reduced cathepsin B levels (Supplementary Figure 8B). These results indicated that, among three MAPKs, only ERK phosphorylation was responsible for reducing cathepsin B levels.

### Bortezomib blocked cisplatin-induced autophagy and enhanced the anticancer effects of cisplatin

**Figure 6:**
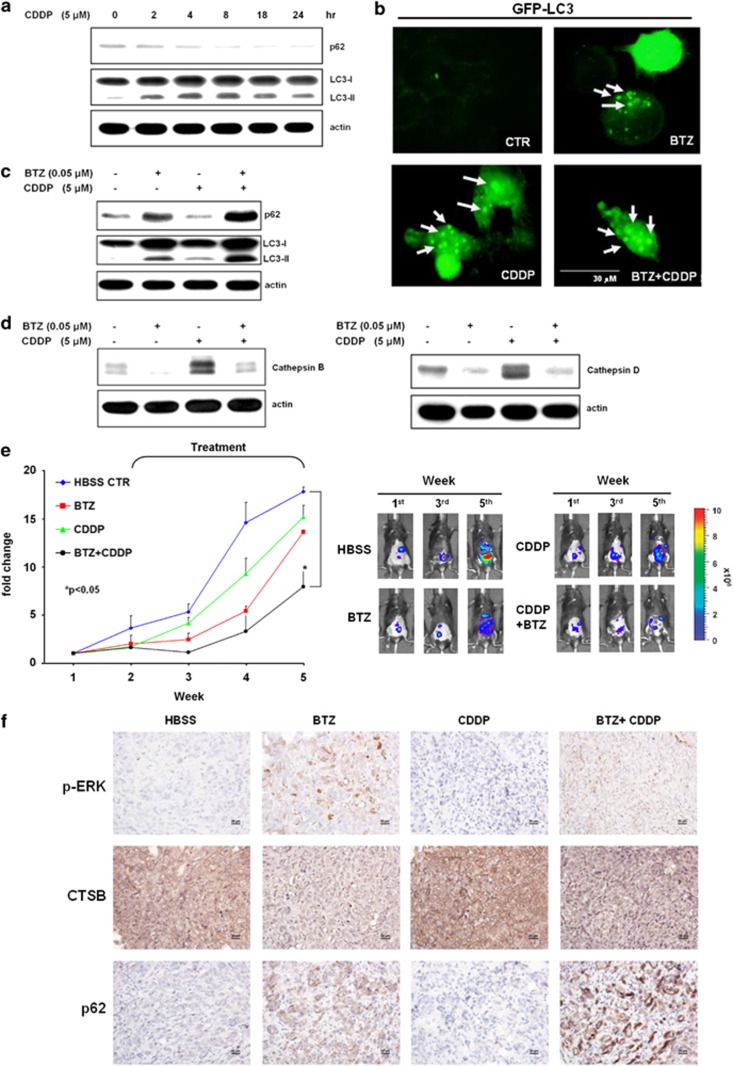
Bortezomib (BTZ) blocks cisplatin (CDDP)-induced autophagy and enhances cytotoxicity in mice. (**a**) Treatment with 5 *μ*M CDDP caused a time-dependent autophagy of TOV112D, shown by increasing levels of LC3-II and decreasing levels of p62. (**b**) Puncta formation (arrow) in TOV112D cells treated with 0.05 *μ*M BTZ, 5 *μ*M CDDP, or the combination of both for 24 h. Scale bar represents 30 *μ*m. (**c**) Treatment with 0.05 *μ*M BTZ inhibited CDDP-induced autophagy, shown by the increased level of p62 and LC3-II. (**d**) CPPD-induced autophagy was characterized with increased levels of cathepsin B, which was inhibited by the co-treatment with BTZ. (**e**) Ten millions of MOSEC/LUC cells were intraperitoneally injected into C57BL/6 mice. Subsequently, the mice were intraperitoneally injected with 100 *μ*L HBSS (vehicle alone), 20 *μ*g/mL BTZ per mouse, 100 *μ*L CDDP (80 *μ*M) per mouse, or both twice a week. The mice were then analyzed with the IVIS 200 *in vivo* imaging system on a weekly basis. (**f**) Protein levels of p-ERK, cathepsin B (CTSB), and p62 from representative tumors from the aforementioned studied mice were analyzed with immunohistochemistry. Protein levels were shown as different intensities of brown color. Cell nuclei were counterstained with hematoxylin. Scale bar represents 20 *μ*m. CTR, control

**Figure 7:**
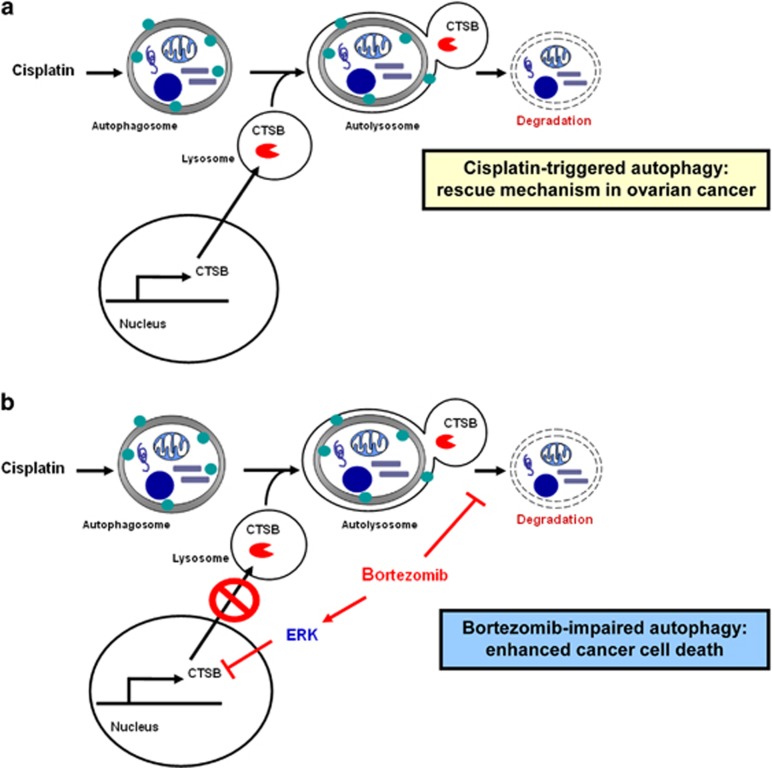
ERK phosphorylation induced by bortezomib inhibits the catalytic process of autophagy and enhances cisplatin-induced cytotoxicity of cancer cells. (**a**) Upon the treatment with cisplatin, cancer cells often use autophagy as a form of self-rescue. Increased levels of cathepsin B (CTSB) are required to complete the catabolic process of autophagy. (**b**) Treatment with bortezomib induces phosphorylation of ERK, which decreases CTSB levels and blocks the catabolic process of autophagy. By adding bortezomib to cisplatin-based chemotherapy, self-rescuing autophagy of cancer cells is suppressed, resulting in an enhanced anticancer efficacy

Cisplatin chemotherapy has been shown to induce autophagy,^32^ which is evolved as a self rescuing mechanism by ovarian cancer cells. So we tested whether the bortezomib-blocked catalytic process of autophagy could sensitize the anticancer effect of cisplatin (CDDP). Cisplatin-stimulated autophagy was shown by a time-dependent increase of LC3-II and concomitant decrease of p62 levels (Figure 6a) as well as an increase of GFP-LC3 puncta formation (Figure 6b). Combination of bortezomib and cisplatin increased GFP-LC3 puncta formation and p62 levels in multiple cancer cell lines (Figure 6b and c, and Supplementary Figure 8). Of note, bortezomib attenuated cisplatin-induced cathepsin B/D (Figure 6d). The *in vivo* treatment with both bortezomib and cisplatin inhibited tumor proliferation more effectively than bortezomib alone (Figure 6e). Immunohistochemical analysis of tumors treated with various reagents also provides *in vivo* evidence that bortezomib activated ERK phosphorylation, inhibited cathepsin B, and inhibited p62 degradation (Figure 6f). Cisplatin-induced autophagy was counteracted by the concomitant treatment with bortezomib, shown by decreased cathepsin B (Figure 6) and increased p62 levels (Figure 6 and Supplementary Figure 9). Collectively, these results suggest a novel strategy to reduce autophagy-related, chemotherapeutic resistance and the clinical potential for the combination of bortezomib and cisplatin in the treatment of ovarian cancer (Figure 7).

## Discussion

Bortezomib has been proposed to stimulate autophagy,^12, 13, 14, 15, 16, 17, 37, 38, 39^ but contrary findings also exist.^9, 40^ Autophagy proteins have been shown to regulate the functions of ERK,^41^ whereas all MAPKs, including ERK,^42^ JNK,^43, 44^ and p38,^38^ have been reported to modulate autophagy.^43, 44^ On the other hand, sustained activation of ERK was shown to inhibit the maturation step of the autophagy process.^45^ Adding more surprises to the complexity among bortezomib, ERK activation, and autophagy, this study appears to be the first one revealing that bortezomib blocks the autophagic flux via the phospho-ERK-mediated reduction of cathepsin B.

Even with current methods for detecting autophagosomes and the LC3 conversion from LC3-I to LC3-II, it remains a challenge to accurately measure the flux of autophagy.^46^ Decreasing p62/SQSTM1 levels concomitant with LC3 conversion have been proposed to be useful in monitoring autophagic flux.^23^ Therefore, we used the p62 change after bortezomib treatment as a criterion to review the reports in which bortezomib was claimed to stimulate autophagy. The levels of p62 were not shown in six reports.^12, 13, 14, 15, 16, 39^ In three reports where p62 levels were shown, bortezomib treatment induced p62 degradation in myeloid leukemic cells,^37^ hepatocellular carcinoma,^17^ and lymphoma cells,^38^ suggesting that bortezomib activates autophagy. However, our findings clearly indicated that bortezomib treatment blocks the autophagic flux in multiple ovarian cancer cell lines, where the numbers of autophagosome and LC3 puncta accumulated and p62 failed to be degraded. Our results are in accordance with the results found with breast cancer cells^9^ and B-Raf-mutated melanoma cells.^40^ We also found that bortezomib treatment resulted in p62 accumulation in hepatocellular carcinoma HepG2 cells and endometrial cancer Ishikawa cells, indicating that autophagic flux was blocked by bortezomib. Taken together, the blockade of autophagic flux appears to be a common effect of bortezomib, at least in solid tumors.

The interactions between ERK activity and autophagy are complex. Autophagy-related protein ATG7 and ATG5 may serve as cellular scaffolds to stimulate ERK phosphorylation.^41^ P62/ SQSTM1 was also shown to inhibit ERK phosphorylation, and the knockout of p62 was shown to enhance ERK activation *in vivo*.^47^ On the other hand, ERK knockdown has been shown to induce autophagy,^48^ and sustained ERK activation may inhibit the autophagic flux.^45, 49^ Our results that bortezomib induced ERK phosphorylation was similar to those reported by Codony-Servat and colleagues.^50^ Our findings that ERK phosphorylation reduced cathepsins further explain how ERK phosphorylation inhibits the autophagic flux.

Cathepsins are lysosomal proteases required for autophagic degradation processes,^51^ and they are important for the death, proliferation, and invasion of human cancer cells.^36^ Cathepsins are overexpressed in many carcinomas and frequently associated with poor clinical prognoses.^52, 53^ We found that bortezomib decreased the activity of lysosomal degradation without inhibition of the fusion between autophagosome and lysosome. Our findings were consistent with the report that bortezomib reduces cathepsin activity to block autophagy and decreases cell proliferation.^9^ Our study also provides an additional role of ERK in the inhibition of autophagy. By phosphorylating serine 142, ERK inhibits TFEB that drives the expression of autophagy and lysosomal genes.^48^ Thus, ERK may keep autophagy in check at the transcriptional level. We also found that ERK activation induced by bortezomib can also inhibt the lysosomal stage of autophagy by suppressing cathepsins.

Upon exposure to cytotoxic therapy such as cisplatin, normal and cancer cells activate autophagy to protect themselves from damage.^54^ Therefore, the combination of chemotherapy with an autophagy-inhibiting mechanism has been proposed to be a viable strategy for cancer therapy.^46^ Although platinum-based chemotherapy is the therapeutic mainstay of ovarian cancer treatment,^33^ increased autophagy was observed in cisplatin-resistant ovarian cancer cells.^55^ Blockade of autophagy with 3-methyladenine has recently been shown to be effective in cisplatin-based chemotherapy for ovarian cancer cells.^56^ Likewise in our study, the addition of bortezomib blocks cisplatin-activated autophagy, and both drugs synergistically kill cancer cells *in vitro* and *in vivo* (Figure 6). Currently, clinical trials are investigating the use of the combination of bortezomib and cisplatin in the treatment of ovarian cancer.^52^

In conclusion, the impact of autophagy in different cancers upon anticancer therapy is apparently context-dependent.^26^ At least in many solid tumors, bortezomib may induce ERK phosphorylation to suppress cathepsin B, and inhibit the catalytic process of autophagy. The inhibition of cisplatin-induced autophagy by bortezomib can enhance chemotherapy efficacy in ovarian cancer. As we also found that bortezomib blocks autophagy in endometrial cancer and hepatocellular cancer, the synergistic cytotoxic effect of bortezomib and cisplatin may be an option as an adjuvant therapy option for multiple cancers.

## Materials and Methods

### Cell culture and reagents

Human ovarian cancer cell lines TOV112D, TOV21G, OV90, SKOV3, MDAH2774, and ES2 were obtained from ATCC (Rockville, MD, USA). BR and BG1 cells were described previously.^10^ Cancer cells were cultured in Dulbecco's modified Eagle's medium/F-12 supplemented with 10% fetal bovine serum and antibiotics at 37 ^o^C with 5% CO2. Bortezomib (Millennium Pharmaceuticals, Cambridge, MA, USA) was dissolved in medical injectable water at the concentration of 10 mM. Another proteasome inhibitor MG132 (Sigma, St Louis, MO, USA, M7449-200UL) was dissolved in DMSO at 10 mM. Autophagy inhibitor 3-methyladenine (Sigma, M9281-100MG) was dissolved in medical injectable water at 50 mM. Cycloheximide (Sigma, C1988-1G) and chloroquine (Sigma, C6628-25G) were dissolved in medical injectable water at 10 mM. Rapamycin (Sigma, R8781-200UL) was dissolved in DMSO at 10 mM, and PD98059 (Sigma, P215-1MG) was dissolved in DMSO at 100 mM. Cisplatin was available at 0.5 mg/ml (Fresenius Kabi, Raleigh, NC, USA).

### Cell viability assay

Cells were treated with designated concentrations of bortezomib and 3-methyladenine in Dulbecco's modified Eagle's medium/F-12 supplemented with 10% fetal bovine serum for 24 h before assay. The growth inhibition effect of bortezomib was measured with MTT (3-(4,5-dimethylthiazol-2-yl)-2,5-diphenyltetrazolium bromide) method (Sigma, M5655-1G). TOV112D cells were plated at 10 000 cells/well in 96-well plates. Working concentration of MTT solution was 1 mg/ml. Optical density was measured using VICTOR2 Scanning multi-well spectrophotometer (Bio Surplus, San Diego, CA, USA) with the absorbance at 570 nm.

### Western blot analysis

Cells were lysed in ice-cold RIPA lysis buffer (1% Triton X-100, 1% NP-40, 0.1% SDS, 0.5% DOC, 20 mM Tris-hydroxymethyl-aminomethane (Tris-HCl, pH 7.4), 150 mM NaCl, protease inhibitors (Sigma), and phosphatase inhibitors (Sigma)) for 30 min. Following electrophoretic separation on a 10% SDS-PAGE gels (LC3 on 13% SDS-PAGE gels), the proteins on the gels were transferred to nitrocellulose membranes (Amersham Pharmacia Biotech, Uppsala, Sweden). Protein samples were analyzed using anti-LC3 (Novus Biologicals, Littleton, CO, USA; NB100-2220), anti-ATG5 (Novus Biologicals, NB110-53818), anti-beclin1 (Epitomics, Cambridge, MA, USA; 2026-1), anti-phospho ERK (Cell Signaling, Danvers, MA, USA; 4376), anti-phospho-JNK (Cell Signaling, 4668), anti-phospho-p38 (Santa Cruz Biotechnology, Dallas, TX, USA; sc-7973), anti-phospho-cJun (Cell Signaling, 3270), anti-p62 (PROGEN Biotechnik, Heidelberg, Germany; GP62), or anti-cathepsin B (Biovision, Milpitas, CA, USA; 3190-100) as primary antibodies, and corresponding horseradish peroxidase-conjugated secondary antibodies (Santa Cruz Biotechnology, sc-2004, sc2005) or peroxidase-conjugated Affinipure donkey anti-guinea pig IgG (secondary antibody for p62) (Jackson ImmunoResearch Laboratories, West Grove, PA, USA; 706-035-148). Labeled proteins were subsequently detected by enhanced chemiluminescence (ECL, Millipore, Bradford, MA, USA). For each sample, band intensities were normalized to *β*-actin (Sigma, sc-47778).^57,58^

### RNA interference

For shRNA transfection, 3 × 10^6^ cells were resuspended in 300 *μ*l of RPMI1640, and cell suspensions were mixed with 30 *μ*g of shRNA. We used electroporation to transfect shRNA at 100 voltages for 70 mSec with ECM2001 instrument (BTX Instrument Division Harvard Apparatus, Inc., Holliston, MA, USA). The shRNA corresponding to the human cDNA sequence for BECN1 (5-CCCGTGGAATGGAATGAGATT-3) were purchased from National RNAi Core Facility, Academia Sinica, Taiwan.

### DNA transfection

The analyses of sh-BECN1, GFP-LC3 (a gift from Dr. Jennifer Leppincott-Schwartz, National Institutes of Child Health and Human Development, Bethesda, MD, USA), C9-CTSB and GFP-RFP-LC3 (obtained from Addgene Inc., Cambridge, MA, USA), GFP-active ERK (Y205D) were described previously.^59,60^ For transfection experiments, 3 × 10^6^ of TOV112D or TOV21G cells were resuspended in 300 *μ*l of RPMI1640, and cell suspensions were mixed with 30 *μ*g of DNA. We used electroporation to transfect shRNA at 100–120 voltages for 70 mSec with the ECM2001 instrument (BTX Instrument Division Harvard Apparatus, Inc., Holliston, MA, USA).

### Immunofluorescent microscopy

After transient transfection with GFP-LC3 or GFP-RFP-LC3, cancer cells were cultured on chamber slide at the concentration of 10^3^ cells per well overnight. After treatment with bortezomib overnight, cells were fixed with acetone for 5 min and incubated in blocking buffer (5% normal goat serum in PBS) for 1 h at RT to reduce nonspecific binding. For LAMP-2 (Sigma, PRS3627) or cathepsin B (Biovision, 3190-100) staining, cells were incubated with a rabbit polyclonal antibody (1 : 100; Invitrogen, Grand Island, NY, USA; A22283-300L) overnight. After being incubated with Alexa Fluor 546 conjugated anti-rabbit IgG (1 : 100; Invitrogen, A22283-300L), the slides were mounted with mounting medium (SouthernBiotech, Birmingham, AL, USA; 0100-20) and cover glass, and analyzed with the Leica TCS SP2 laser-scanning confocal system (Leica, Wetzlar, Germany) as described previously.^61^

### Immunohistochemistry

Paraffin-embedded tumor tissue sections (4 *μ*m) were deparaffinized with xylene and rehydrated through ethanol gradient series. Sections were stained with an anti-p62 antibody (PROGEN Biotechnik) and second anti-guinea pig IgG antibody (Jackson ImmunoResearch Laboratories, 706-035-148) using an immunohistochemistry stainer equipped with a Ventana Basic DAB (3, 30-diaminobenzidine) Detection Kit (Tucson, AZ, USA) according to the manufacturer's protocol. Hematoxylin was used for counterstaining in all specimens as described previously.^62, 63^

### Transmission electron microscopy

Cancer cells were plated at 1 × 10^6^ cell/well in 24-well plates and treated with designated concentrations of bortezomib for 24 h. After the cells were washed with PBS twice, they were incubated with fixation buffer (3% glutaraldehyde + 2% paraformaldehyde in 0.1 M cacodylate buffer, pH 7.4) at 4 ^o^C for 2 h. Following the removal of fixation buffer, cells were incubated with 0.1 M cacodylate buffer (pH7.4) at 4 ^o^C for 10 min. After incubation with designated concentrations (30, 50, 70, 95%) of alcohol for gradual dehydration, the cells were incubated with alcohol: Epon = 1 : 1 buffer for 7 h for infiltration, and incubated with 100% Epon for embedding. Cells were stained with 4% uranyl acetate for 2 h and citrate for 10 min. Images were captured with TEM (H-7500, Hitachi Co. Ltd., Tokyo, Japan).

### Tumor growth monitoring using an *in vivo* imaging system

One million of mouse ovarian surface epithelial cancer cells (MOSEC/LUC), which constitutively expressed luciferase, in 100 *μ*l of Hank's balanced salt solution were intraperitoneally injected to each C57BL/6 mouse using a 23-gauge needle (Becton Dickson, Franklin Lakes, NJ, USA). After 2–4 days, mice were injected with luciferin intraperitoneally (100 *μ*l of 0.4 mg/ml luciferin; Goldbio, St. Louis, MO, USA; luck-1) for 10 min, and tumor growth was monitored by luciferase activity detected with the Xenogen IVIS 200 *In Vivo* Imaging System (Xenogen Corp., Alameda, CA, USA). All mice were sedated with isoflurane before the imaging. Light outputs were quantified using the LivingImage software (Xenogen Corp.), as previously described.^10^

### Statistical analyses

Statistical analysis was performed using SPSS statistical software, version 17.0 (SPSS Inc., Chicago, IL, USA). Differences between results were analyzed using the Mann-Whitney U-test. All values were expressed as mean±standard error which was calculated from three independent experiments unless indicated otherwise. *P*-value less than 0.05 were considered statistically significant.

## Abbreviations

ATG: autophagy-related (protein)
BTZ: bortezomib
CDDP: cisplatin
CTSB: cathepsin B
ERK: extracellular signal-regulated kinase
LAMP-2: Lysosomal associated membrane protein 2
LC3: light chain 3
LC3-II: cleaved form of LC3
p62/SQSTM1: sequestosome 1/p62
