# Supplementary material for: Bortezomib enhances cancer cell death by blocking the autophagic flux through stimulating ERK phosphorylation
Source: Cell Death Dis. 2014 Nov 6;5(11):e1510–. doi: 10.1038/cddis.2014.468 (PMC4260726; doi:10.1038/cddis.2014.468)
Supplement: Supplementary information [file cddis2014468x1.doc]

Supplementary information for the manuscript:

**Bortezomib enhances cancer cell death by blocking the autophagic flux through stimulating ERK phosphorylation**

Chieh Kao,^1,2#^ Angel Chao,^1#^ Chia-Lung Tsai,^1^ Wei-Chi Chuang,^1,2^ Wei-Pang Huang,^3^ Guang-Chao Chen,^4^ Chiao-Yun Lin, ^1^ Tzu-Hao Wang,*^1,2,5,6^ Hsin-Shih Wang, ^1,7^ Chyong-Huey Lai^1^

^1^Department of Obstetrics and Gynecology, Linkou Medical Center, Chang Gung Memorial Hospital, Taoyuan, Taiwan

^2^Graduate Institute of Biomedical Sciences, College of Medicine, Chang Gung University, Taoyuan, Taiwan

^3^Institute of Zoology, National Taiwan University, Taipei, Taiwan

^4^Institute of Biological Chemistry, Academia Sinica, Taipei 115, Taiwan

^5^Genomic Medicine Research Core Laboratory, Linkou Medical Center, Chang Gung Memorial Hospital, Taoyuan, Taiwan

^6^School of Traditional Chinese Medicine, College of Medicine, Chang Gung University, Taoyuan, Taiwan

^7^Graduate Institute of Clinical Medical Sciences, College of Medicine, Chang Gung University, Taoyuan, Taiwan

# These authors contributed equally to this work.

*Correspondence to:

Tzu-Hao Wang; e-mail : [knoxtn@cgmh.org.tw](mailto:knoxtn@cgmh.org.tw)

**Supplementary Figure Legends**

**Supplementary Figure 1. Bortezomib (BTZ) increased the levels of beclin 1 and ATG5-ATG12**.

Ovarian cancer TOV112D cells were treated with 0.05 μM bortezomib for 24 h and analyzed for the changes in beclin 1 and ATG5-ATG12 levels.

**Supplementary Figure 2. Autophagy activation could still be induced by a high dose of MG132.**

Ovarian cancer cells were treated with either 0.05μM bortezomib (BTZ) or 5 μM MG132 for 24 h and analyzed for the changes in the levels of LC3 and p62.

**Supplementary Figure 3. Autophagy-related proteins regulated by bortezomib (BTZ) in ovarian cancer cells.** Eight ovarian cancer cells were treated with designated concentrations of bortezomib for 24 h, and changes in a panel of proteins were determined with western blot analyses. In our previous study (Kao C et al, Cell Death and Disease 2013), these ovarian cancer cells could be grouped into 3 groups according to their responses to bortezomib: sensitive, intermediate, and resistant. Sensitive ovarian cancer cells (TOV112D, TOV21G) were treated with 0.05 to 0.1 μM bortezomib, intermediate cells (OV90, BG1, ES2, MDAH2774) were with 1 μM, and resistant cells (BR and SKOV3) were with 10 μM. In all of 8 ovarian cancer cell lines, treatment with bortezomib increased p62 levels. In 7 ovarian cancer cell lines, except for BR, bortezomib increased IκB phosphorylation and inhibited the NFκB signaling pathway. In 6 ovarian cancer cell lines, except for ES2 and MDAH2774, bortezomib stimulated ERK phosphorylation. In 5 (displayed in the upper panel) out of 8 cell lines, bortezomib treatment suppressed cathepsin B (CTSB) levels.

**Supplementary Figure 4. Bortezomib (BTZ) - and MG132-induced ubiquitination**

TOV112D cells were treated with bortezomib or MG132 at designated concentrations for 24 h and analyzed for the changes in ubiquitinated proteins for evaluating the proteasome activity.

**Supplementary Figure 5. Autophagy-related proteins regulated by bortezomib (BTZ) in hepatocellular carcinoma and endometrial cancer.**

A. Hepatocellular cancer HepG2 cells were treated with 0.05 μM BTZ for 12, 24 h and analyzed for the changes in the levels of p-ERK, p62, LC3 and cathepsin B (CTSB). B. Endometrial cancer Ishikawa cells were treated with 0.05 μM BTZ for 24, 48 h and analyzed for the changes in the levels of p-ERK, p62, LC3 and cathepsin B (CTSB).

**Supplementary Figure 6. Bortezomib (BTZ) induced** **phosphorylation of ERK to reduce level of cathepsin B (CTSB) and suppressed autophagic catalytic process in ovarian cancer TOV21G cells. A.** Treatment with 0.1µM BTZ for 24 h decreased CTSB at protein and mRNA levels in TOV21G cells. Results shown are mean ± S.E. derived from three independent experiments. **B.** Treatment with 0.1µM BTZ increased p62 level, which was reduced by overexpression of CTSB. **C.** Forced expression of CTSB attenuated BTZ-mediated growth inhibition in TOV21G cells, which were analyzed with MTT assay. Results shown are mean ± S.E. derived from three independent experiments. **D.** TOV21G were treated with 0.1μM BTZ and 30 μM PD98059 for 24 h and analyzed by immunoblotting for ERK phosphorylation. **E.** TOV21G cells were transfected with C9-CTSB (in green) and treated with 0.1µM BTZ. Following lysosomal labeling for 120 min with 300nM Lysotracker (in red), TOV21G were immunostained with anti-C9 antibody. DAPI staining was used to show nuclei.

**Supplementary Figure 7. High concentrations of MG132 induced phosphorylation of ERK but did not inhibit cathepsin B (CTSB). A.** TOV112D cells were treated with 0.05μM bortezomib (BTZ) and designated concentrations of MG132 for 24 h. CTSB, phosphor ERK were determined with western blot analyses. **B.** TOV112D were treated with 0.05 μM BTZ and 5 μM MG132, in the presence or absence of 30 μM PD98059, respectively, for 24 h and analyzed by immunoblotting for CTSB and ERK phosphorylation.

**Supplementary Figure 8. Bortezomib stimulated JNK phosphorylation but inhibited p38 phosphorylation**. **A**. TOV112D cells were treated with 0.05μM bortezomib (BTZ) and analyzed for the changes in the levels of phospho-p38 and phospho-JNK. **B**. TOV112D cells were treated with 0.05μM bortezomib (BTZ) and designated concentrations of SP600125 for 24 h. Cathepsin B (CTSB), phosphor c-Jun, p62 and LC3 were determined with western blot analyses.

**Supplementary Figure 9.** **Bortezomib (BTZ) blocked cisplatin (CDDP)-induced autophagy and enhanced cytotoxicity. A.** Effects of BTZ on CDDP-induced autophagy in ovarian cancer TOV21G cells were measured by immunoblotting to determine the levels of p62 and LC3-II. The combination treatment with 0.1µM BTZ and 5µM CDDP increased p62 and LC3-II. **(B, C)** Mouse ovarian surface epithelial cancer (MOSEC) cells were treated with either CDDP or BTZ (concentration range: 0.01−10 μM) for 24 h and analyzed for the changes in the levels of p62 and LC3. **D.** BTZ blocked CDDP-induced autophagy in MOSEC cells. MOSEC cells were treated with 0.1µM BTZ and 20µM CDDP, and analyzed for the changes in the levels of p62 and LC3.

**Figure S1**

**
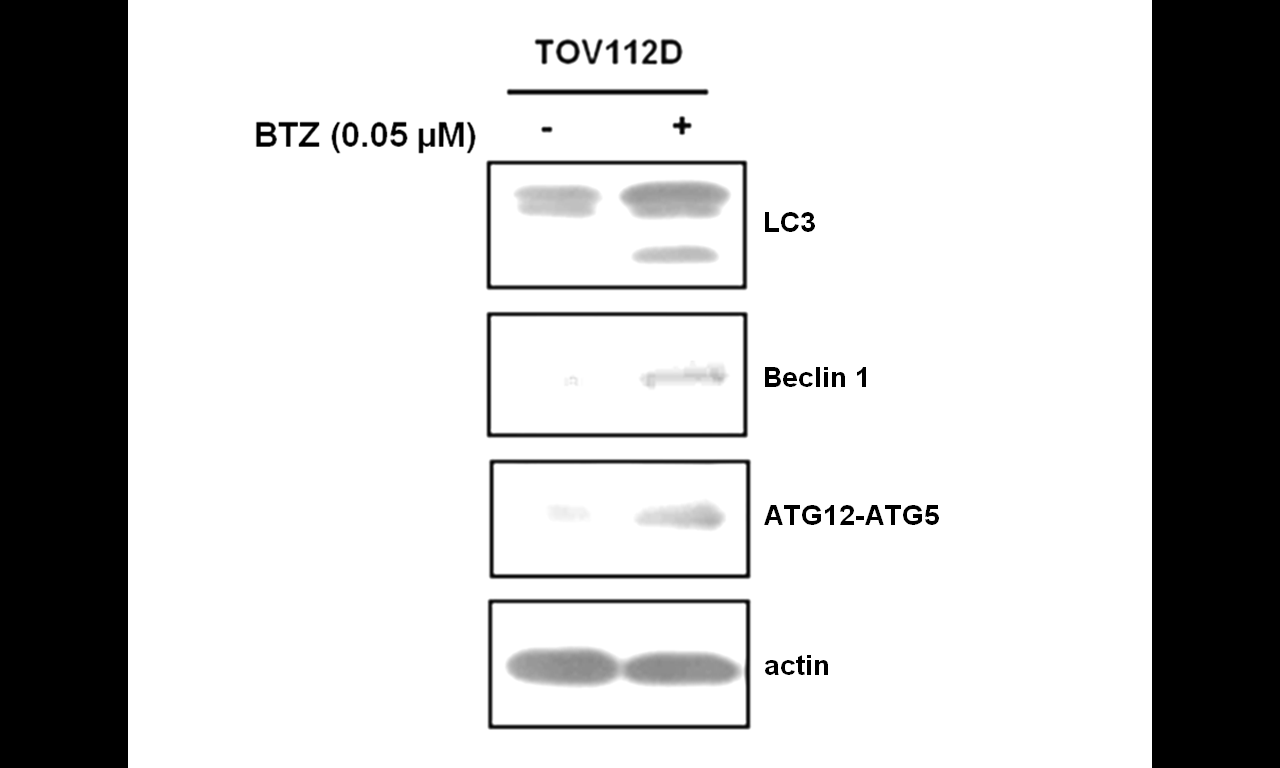
**

**Figure S2**

**
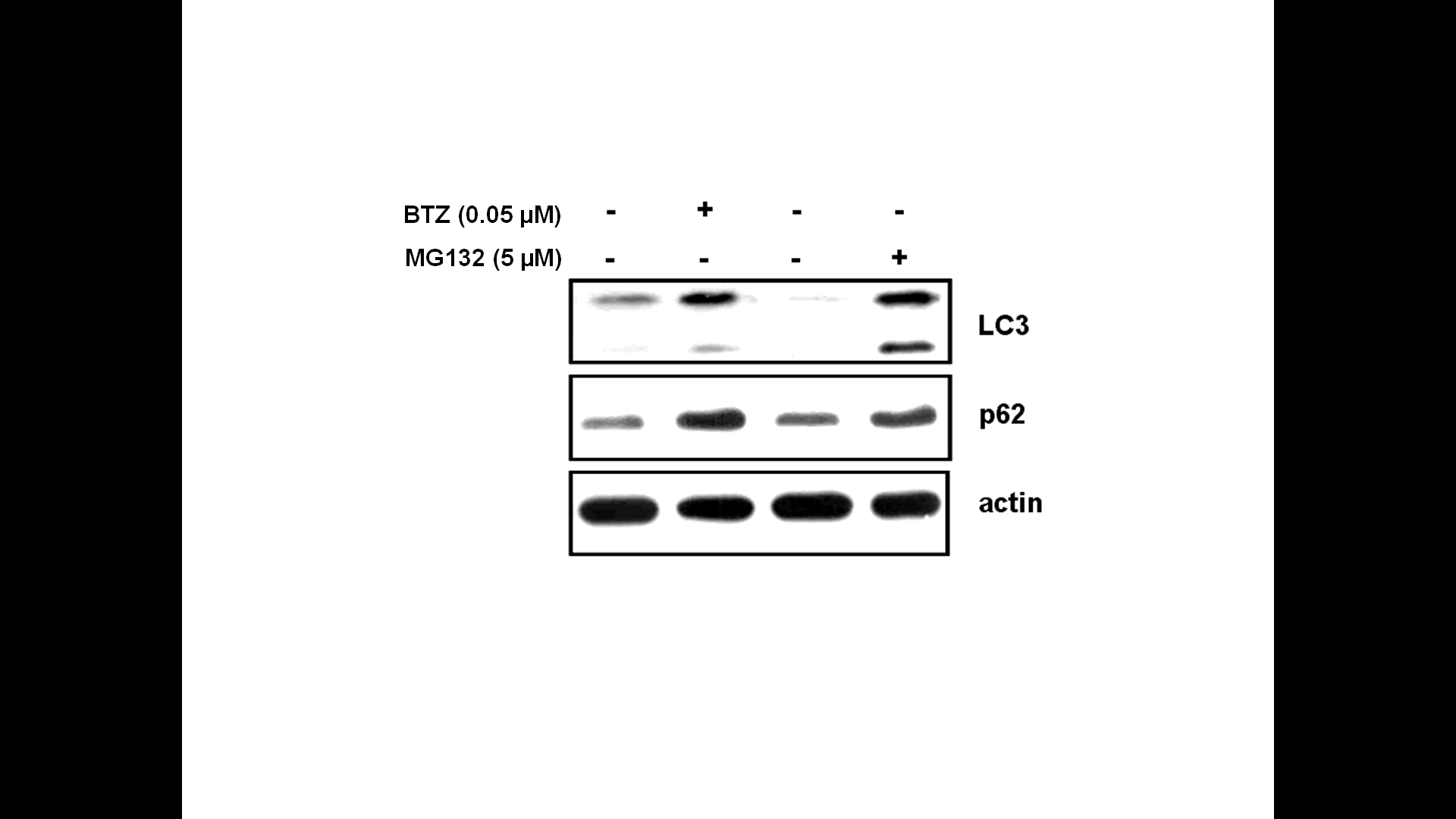
**

**figure S3**


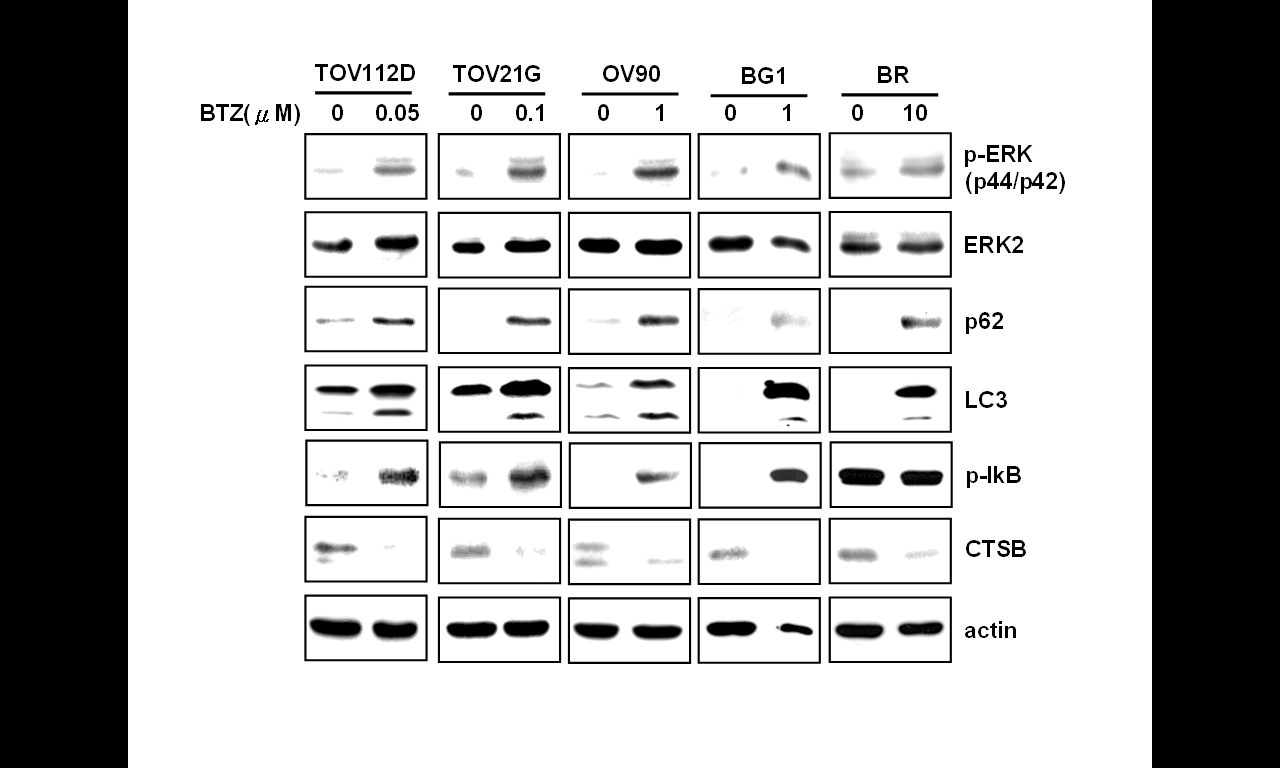


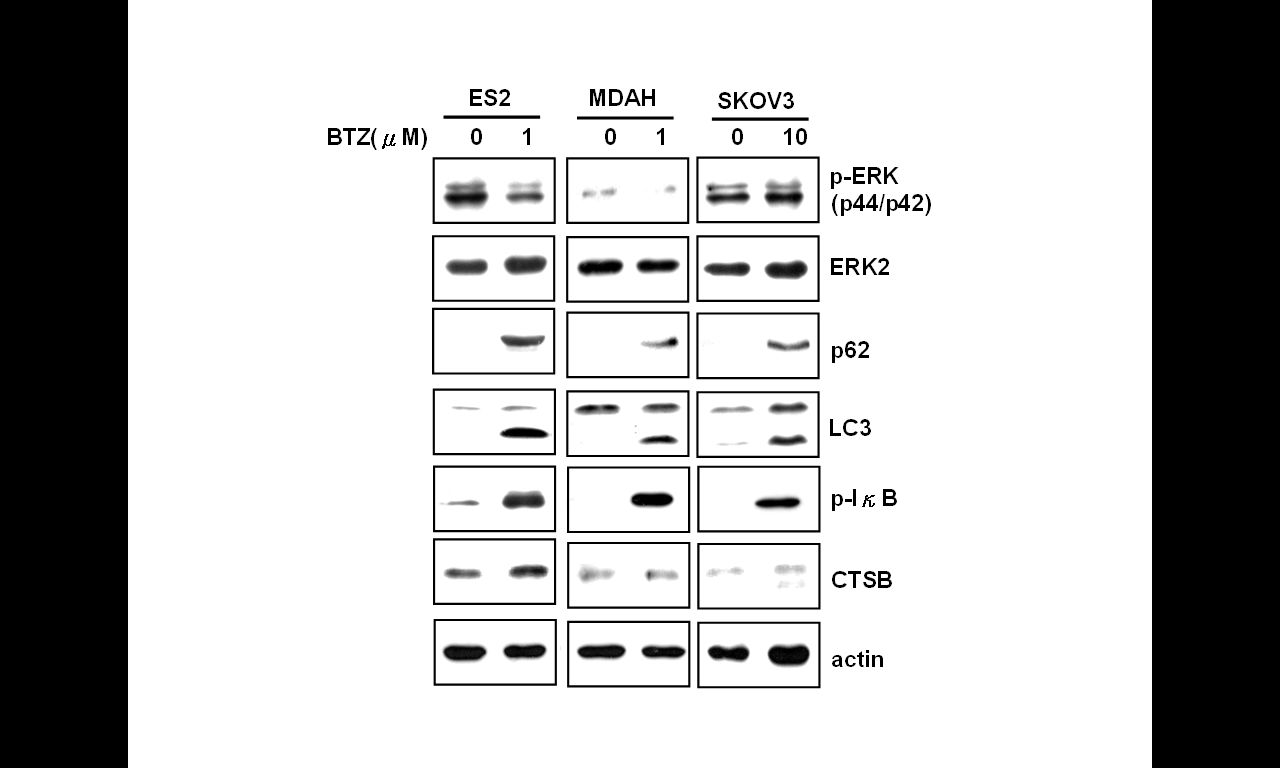


**figure S4**

**
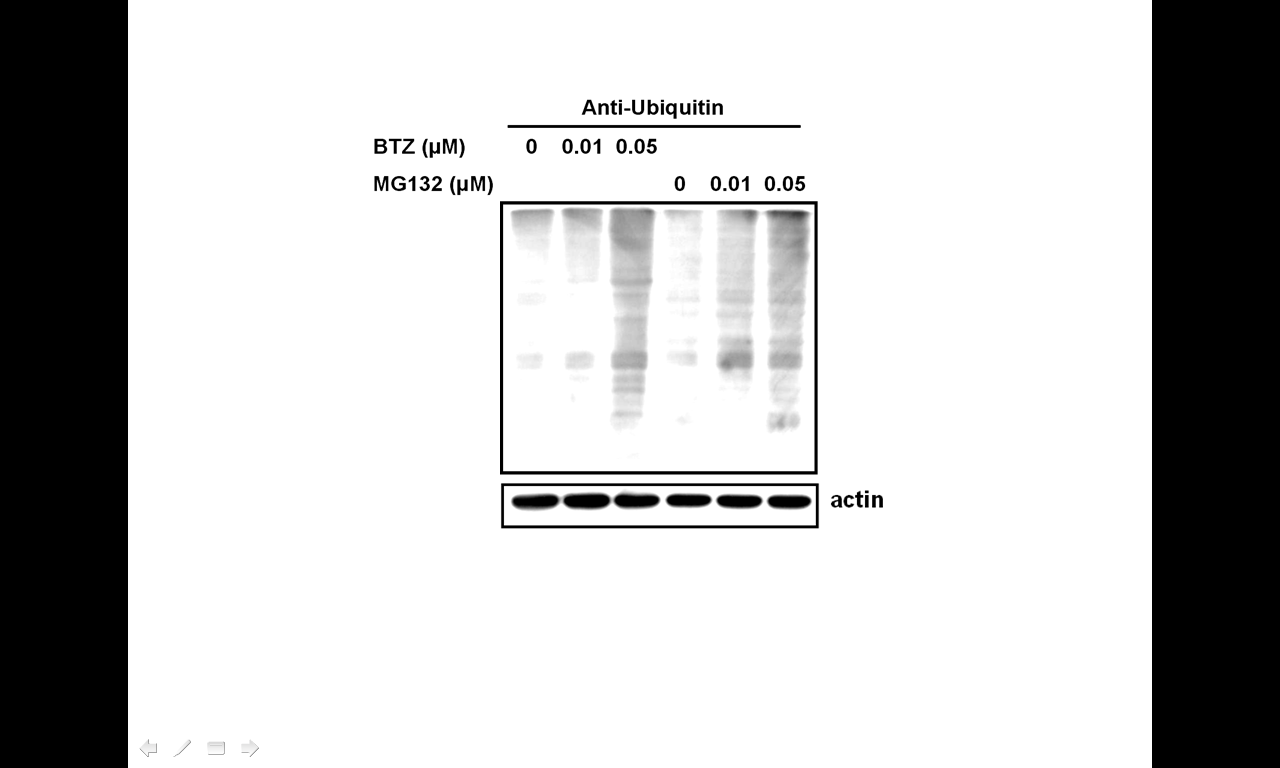
**

**Figure S5**


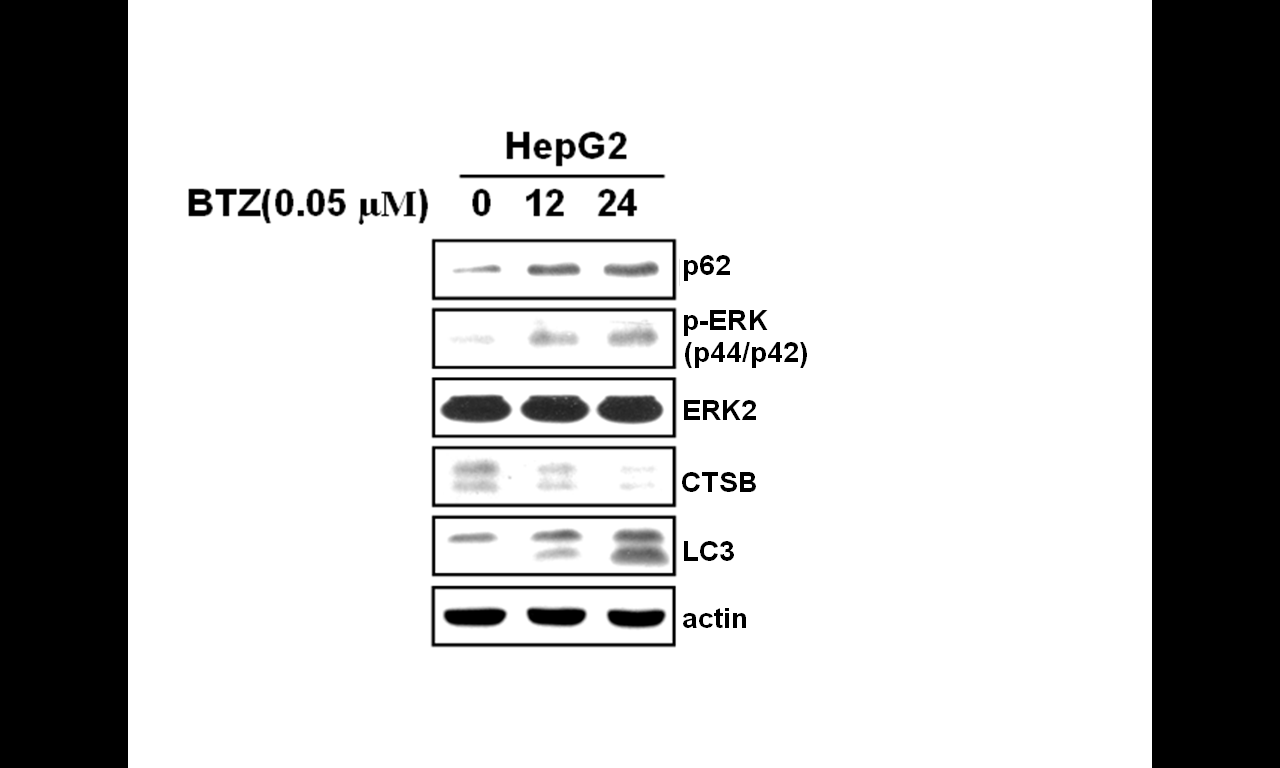
**A**

**B**

**
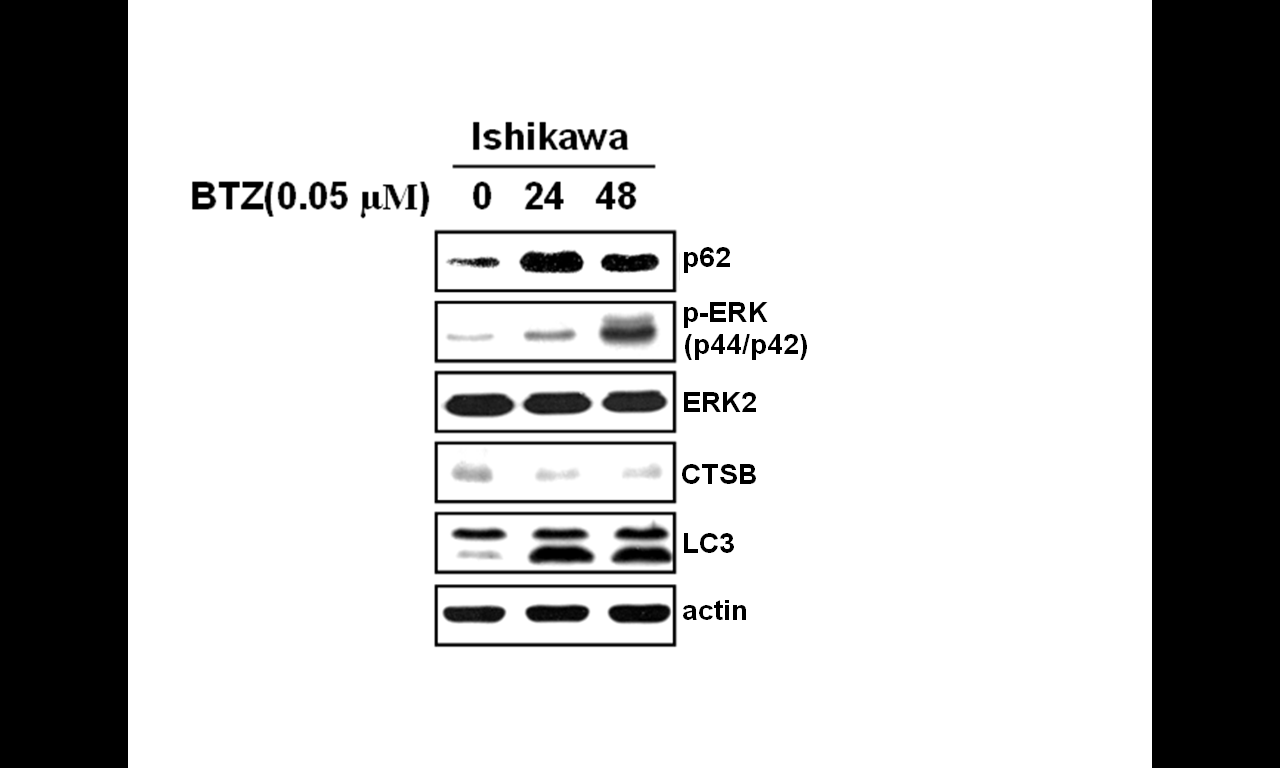
**

**figure S6**

**A B**


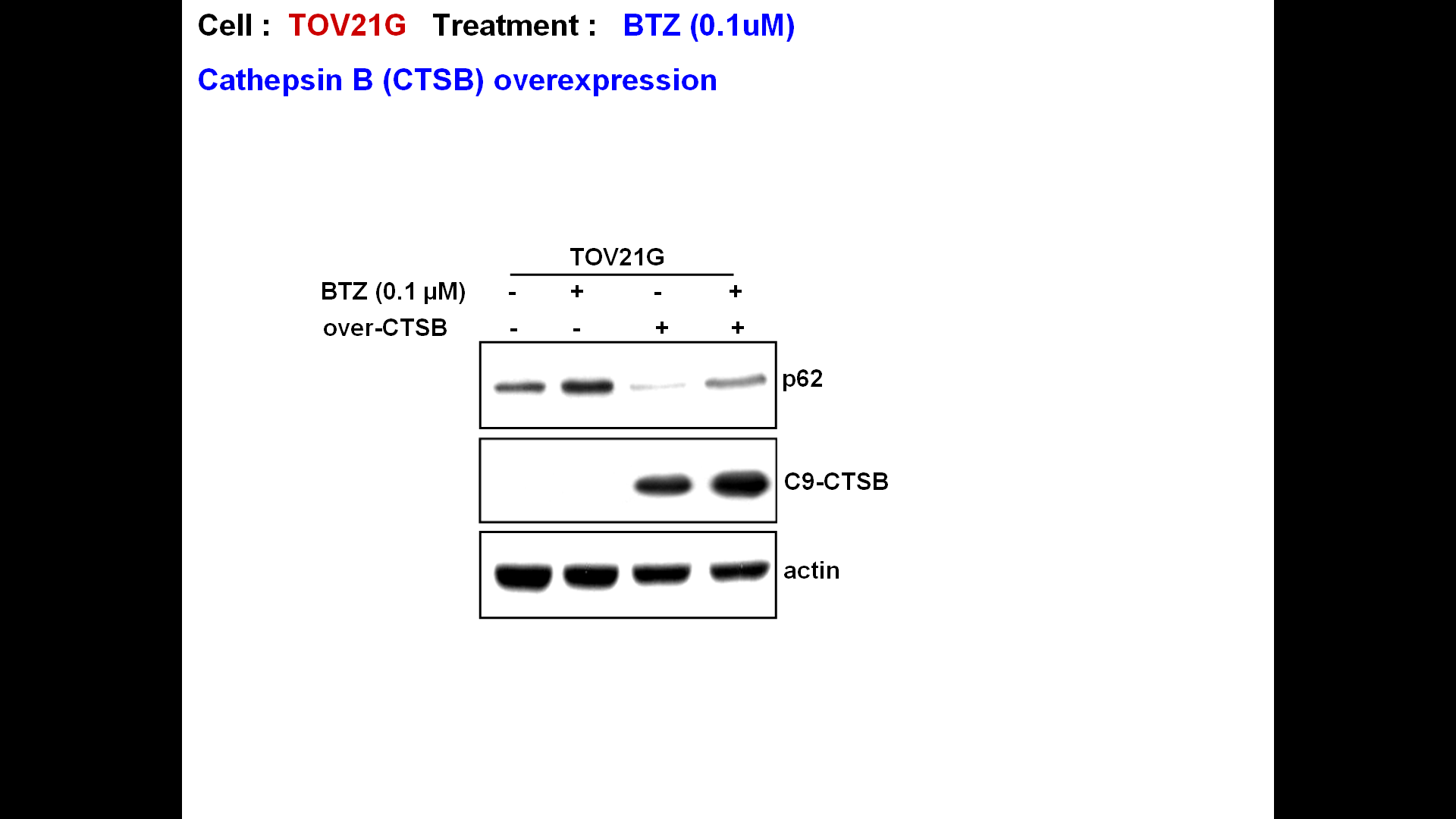

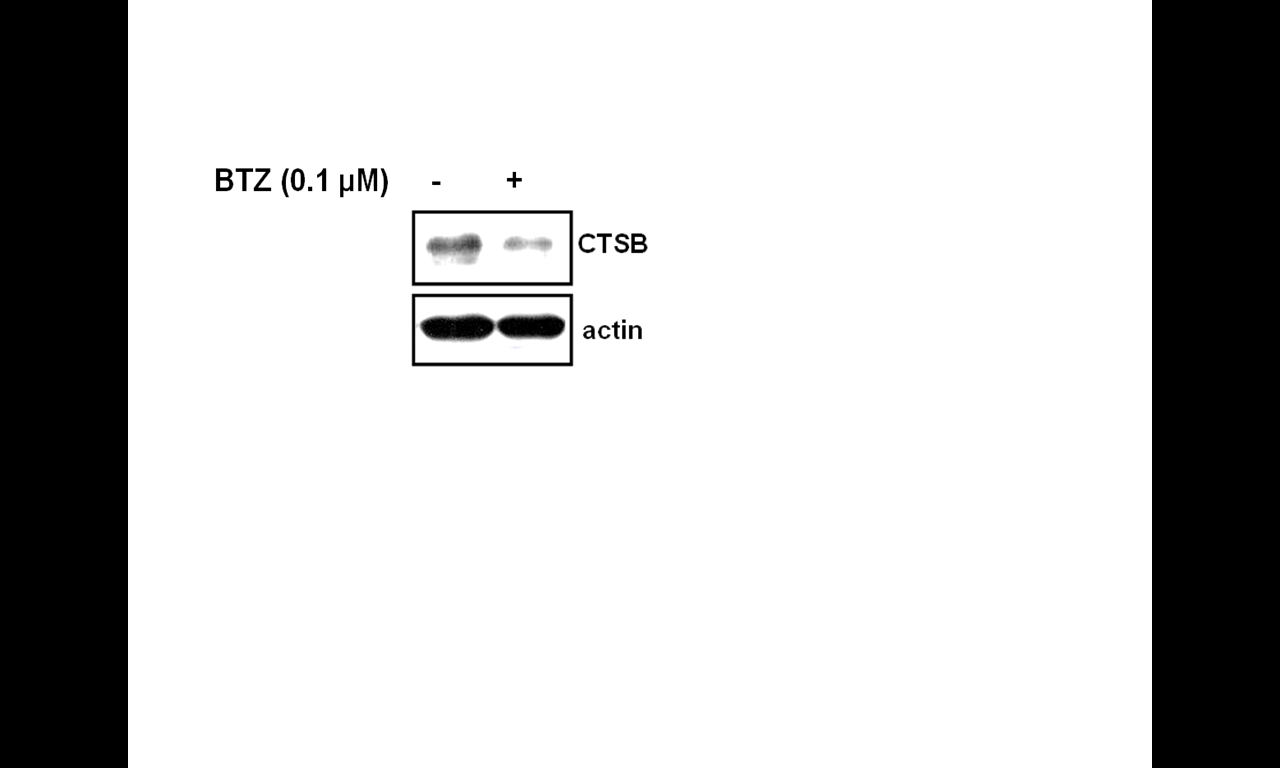


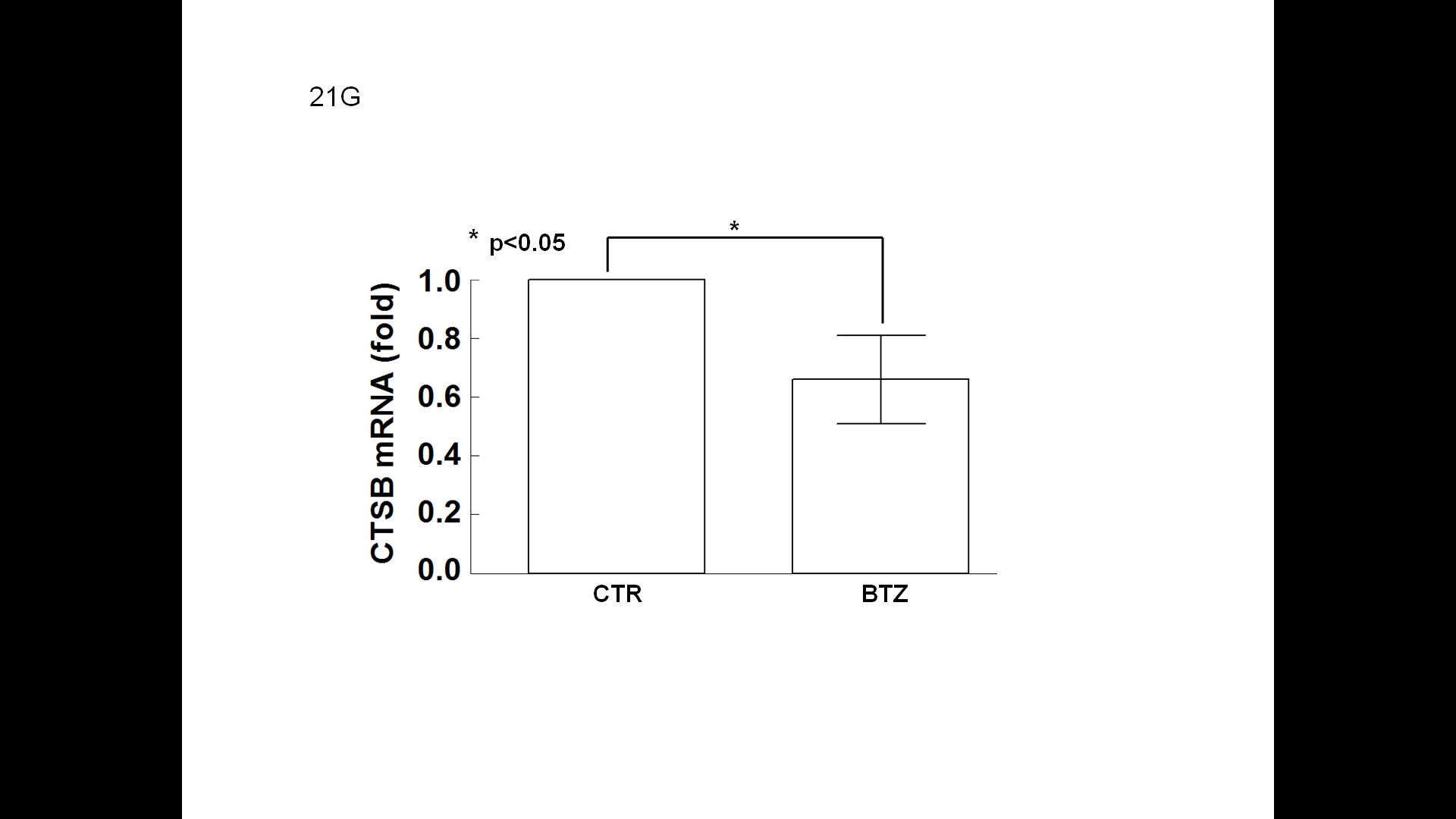


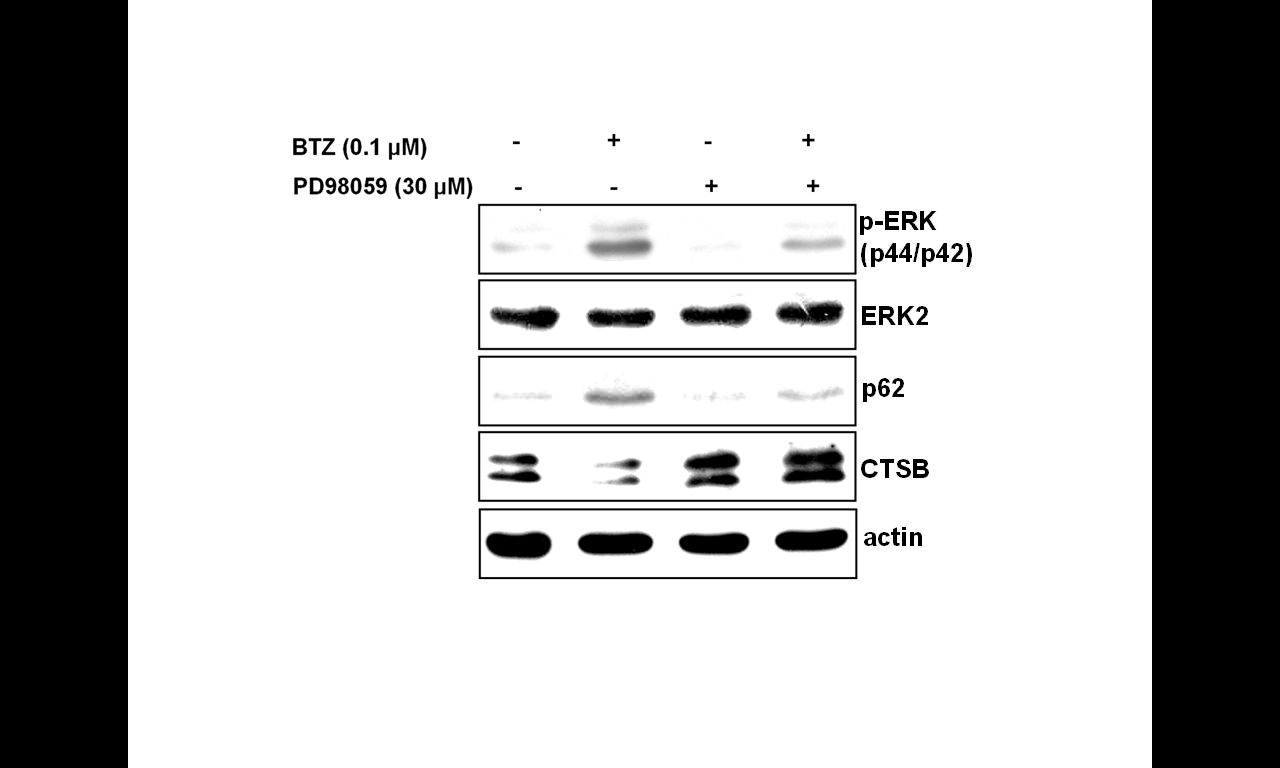


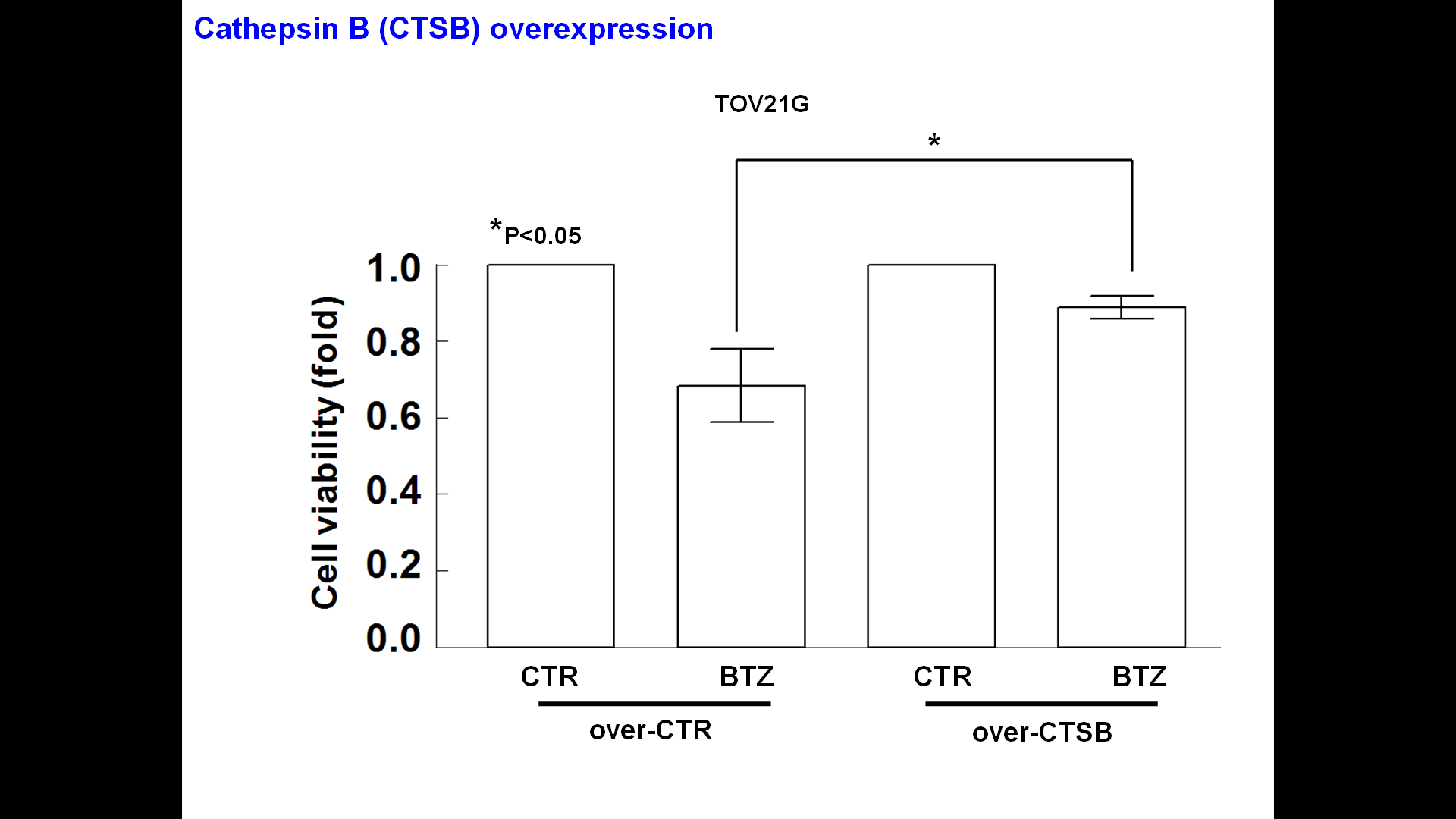
**C D**


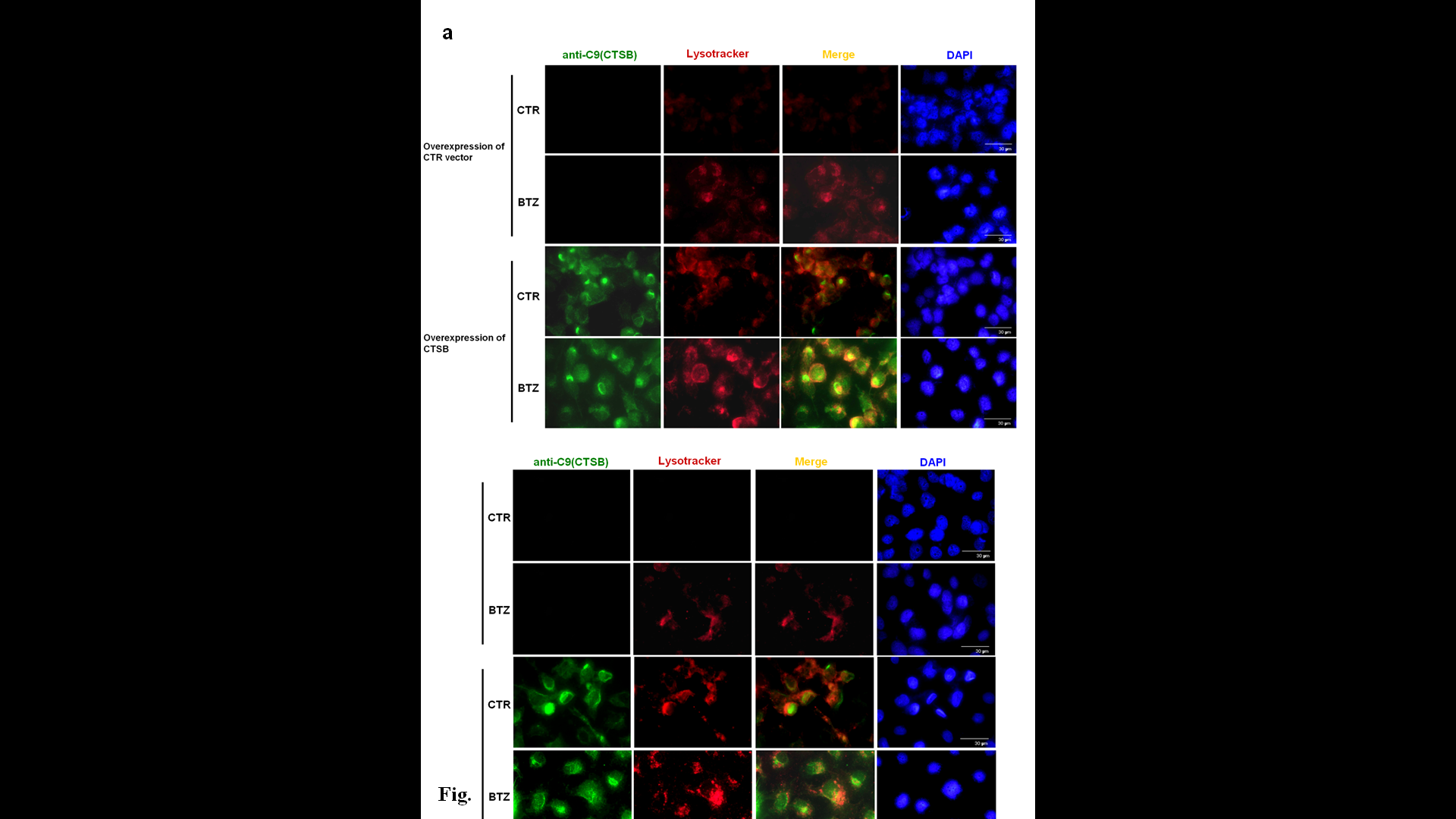
**E**

**figure S7**

A


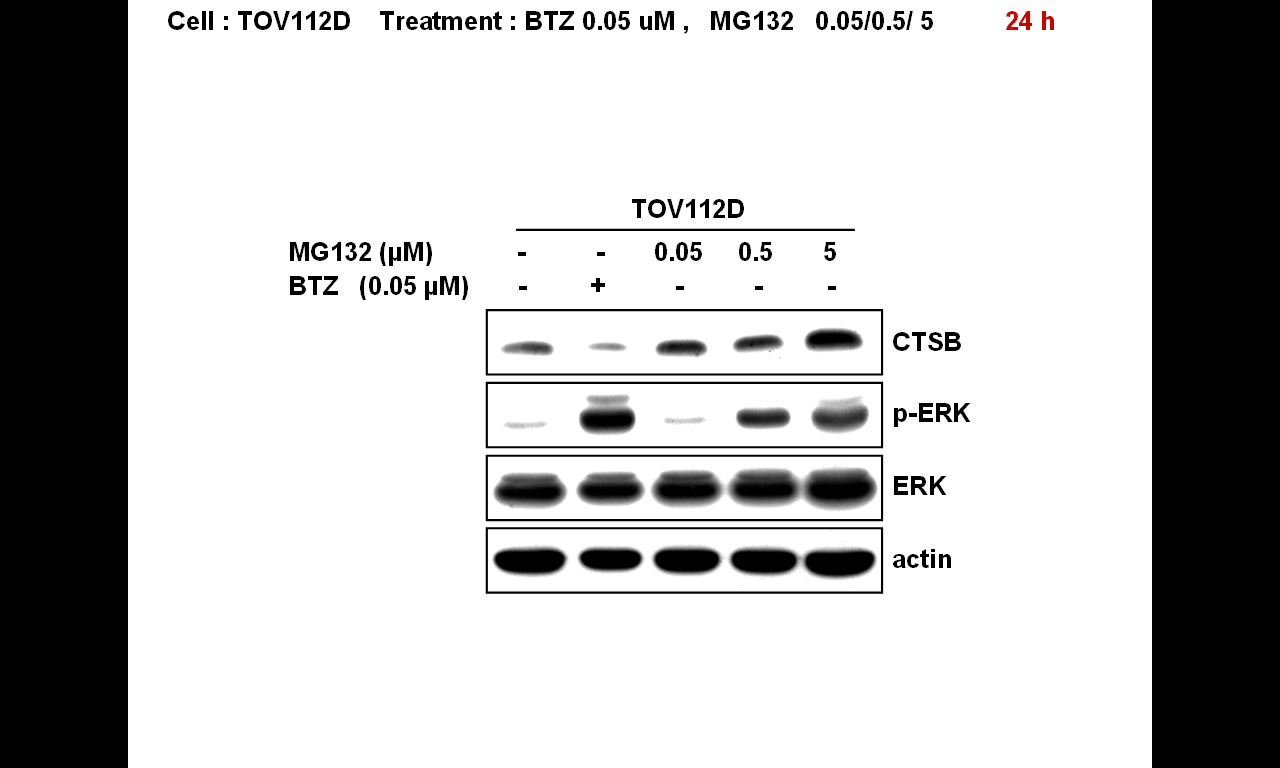


B


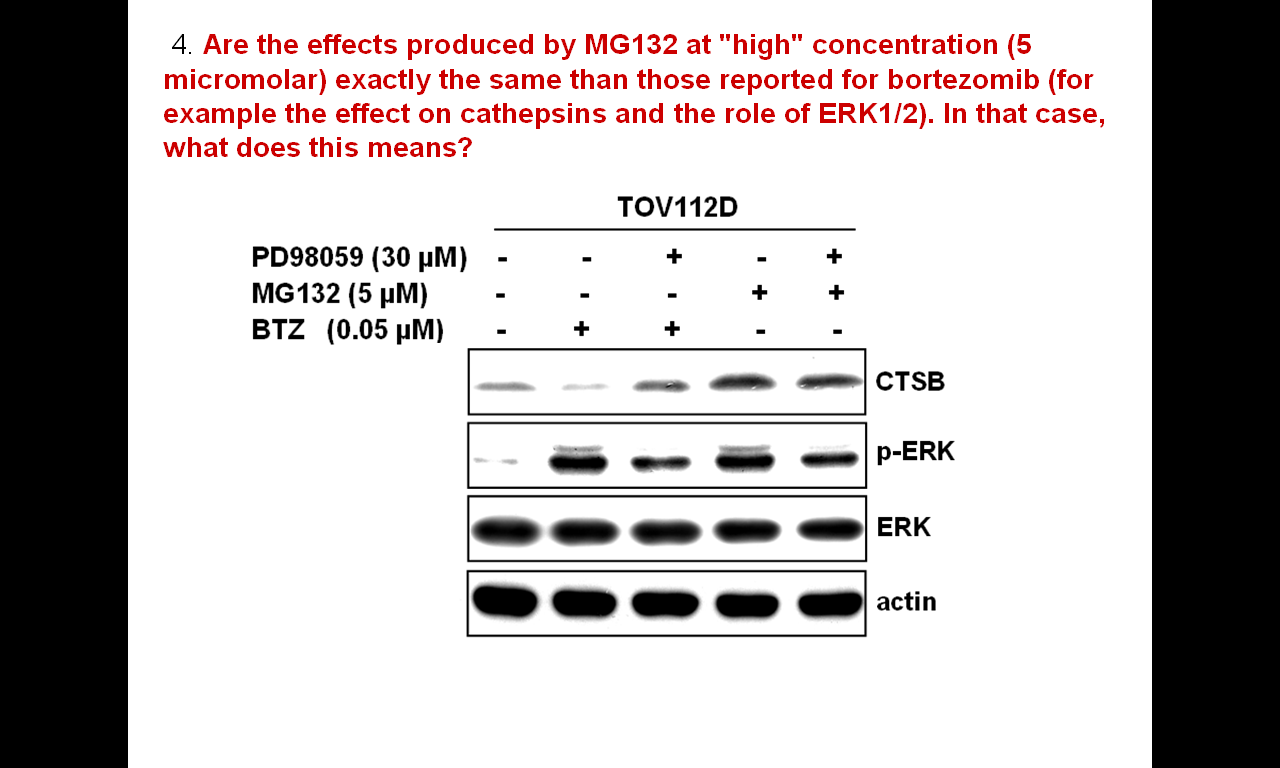


**figure S8**


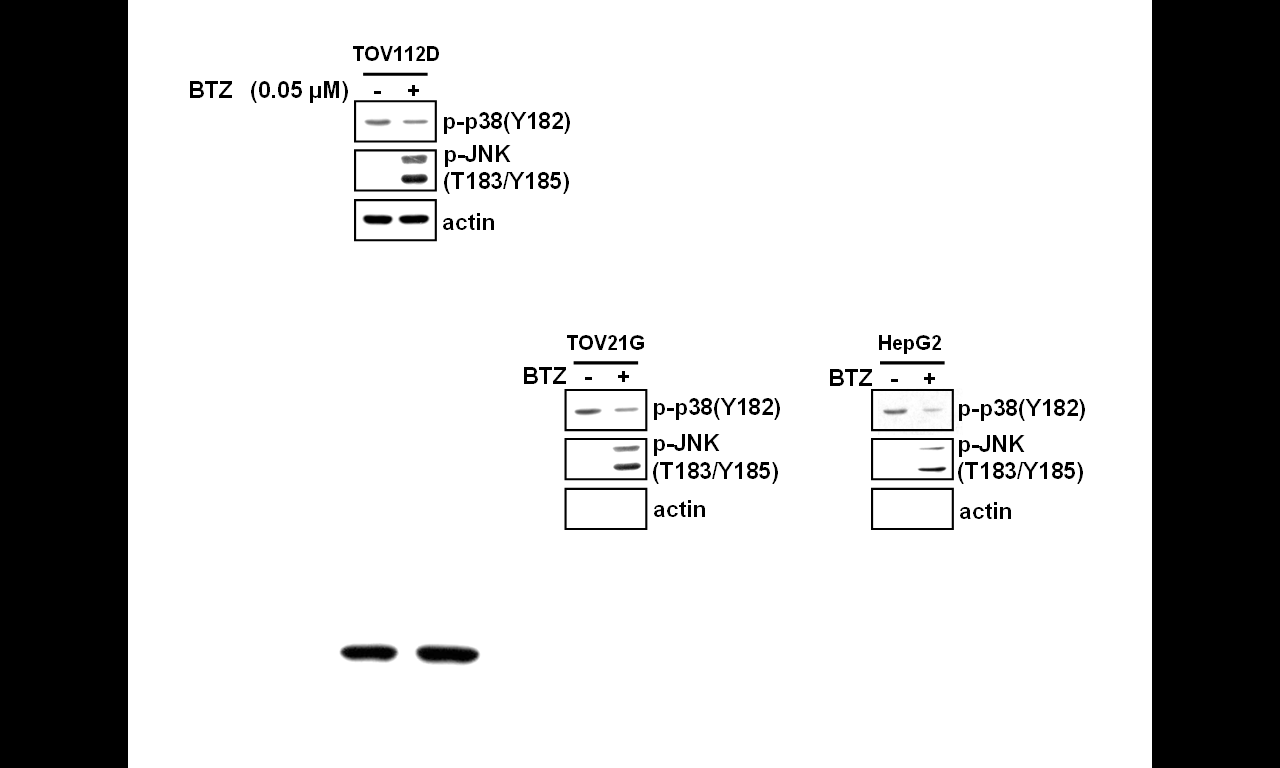
**A**

**B**

**
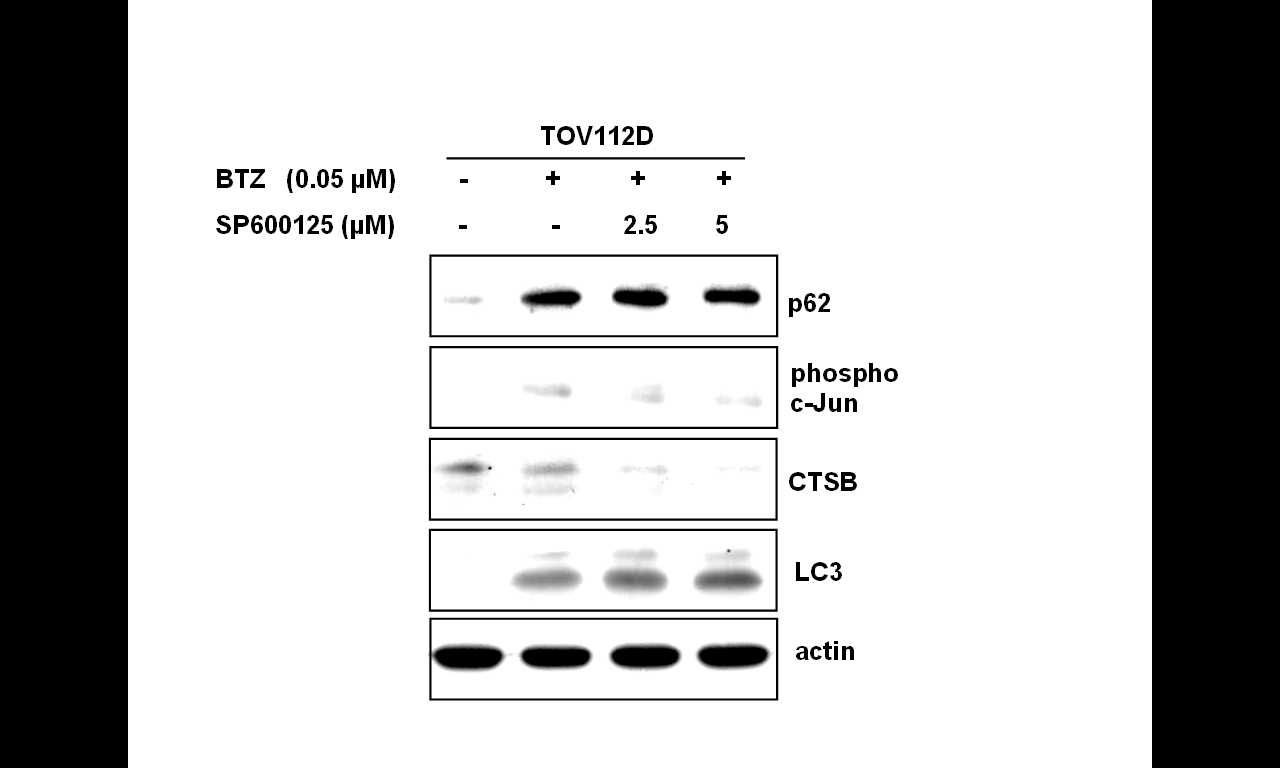
**

**figure S9**


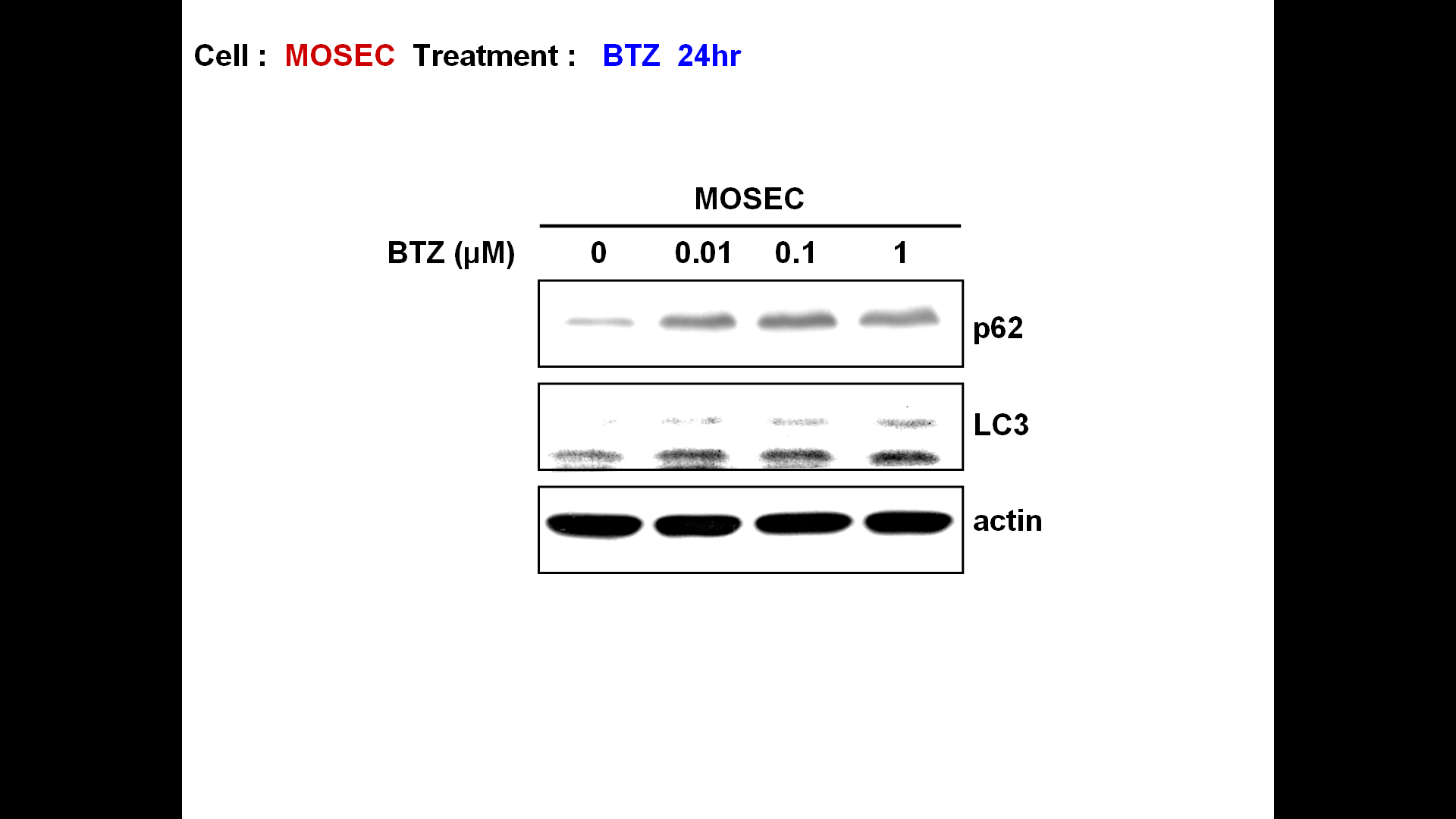

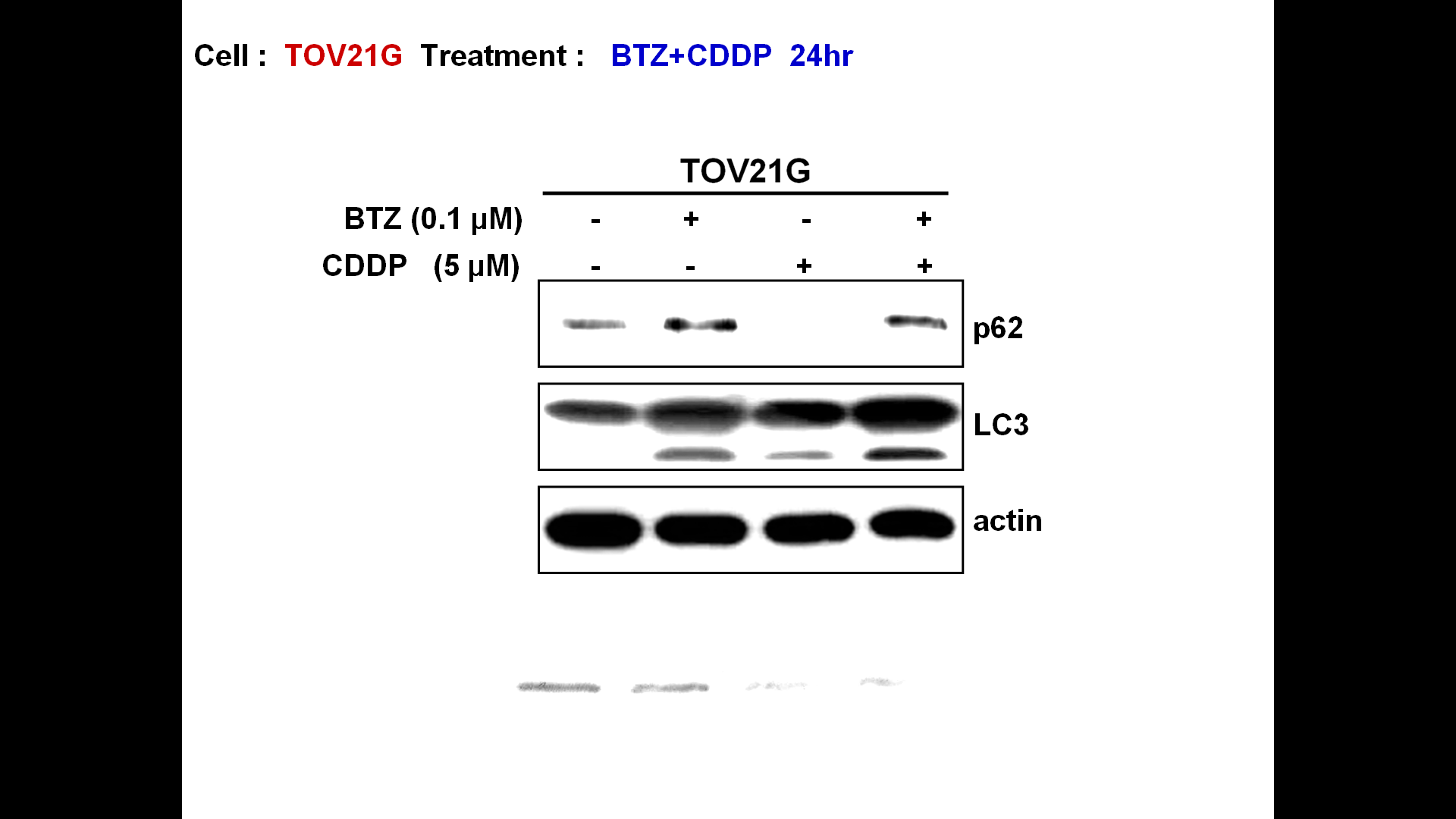
**A B**

**C D**

**
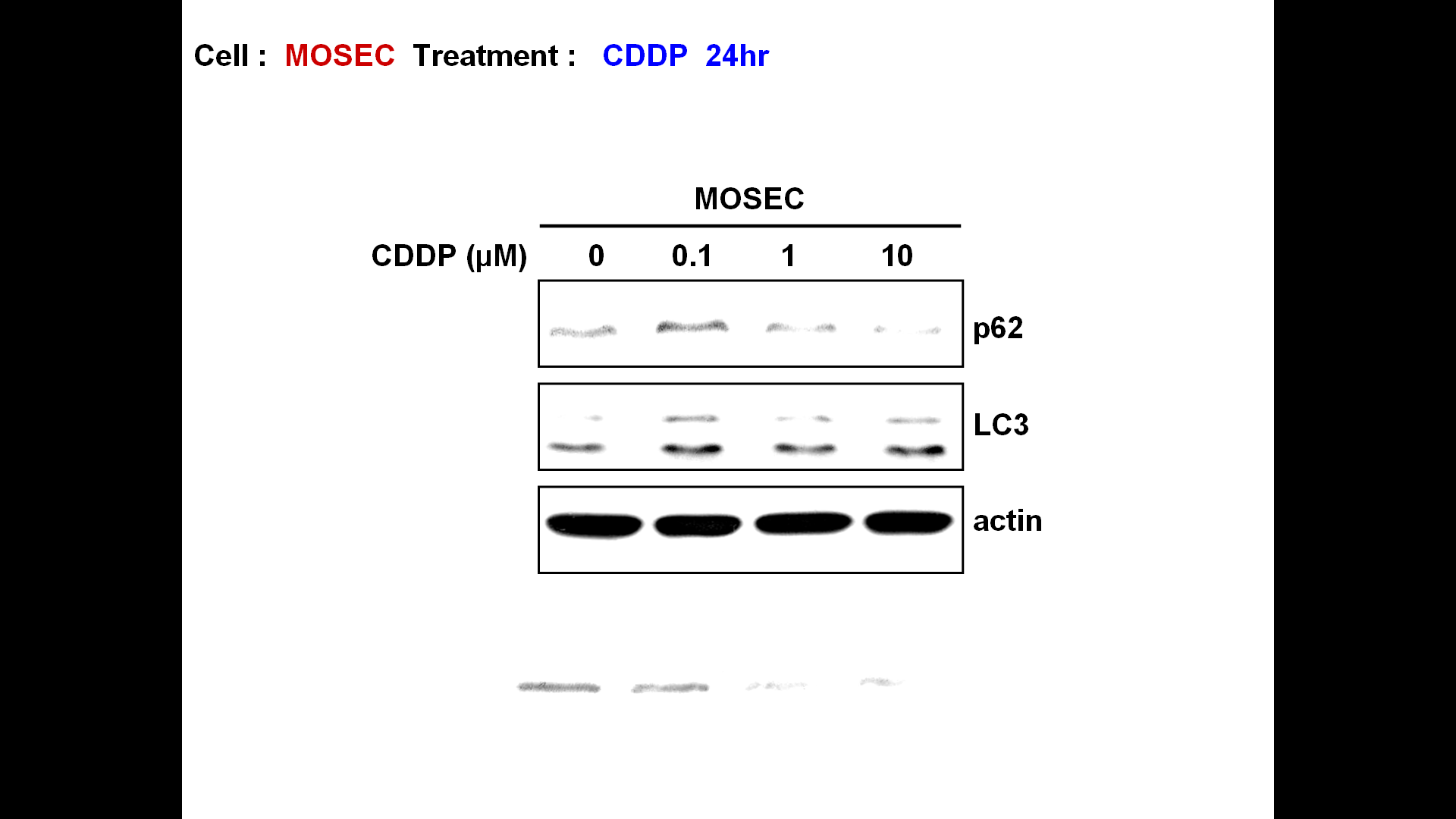

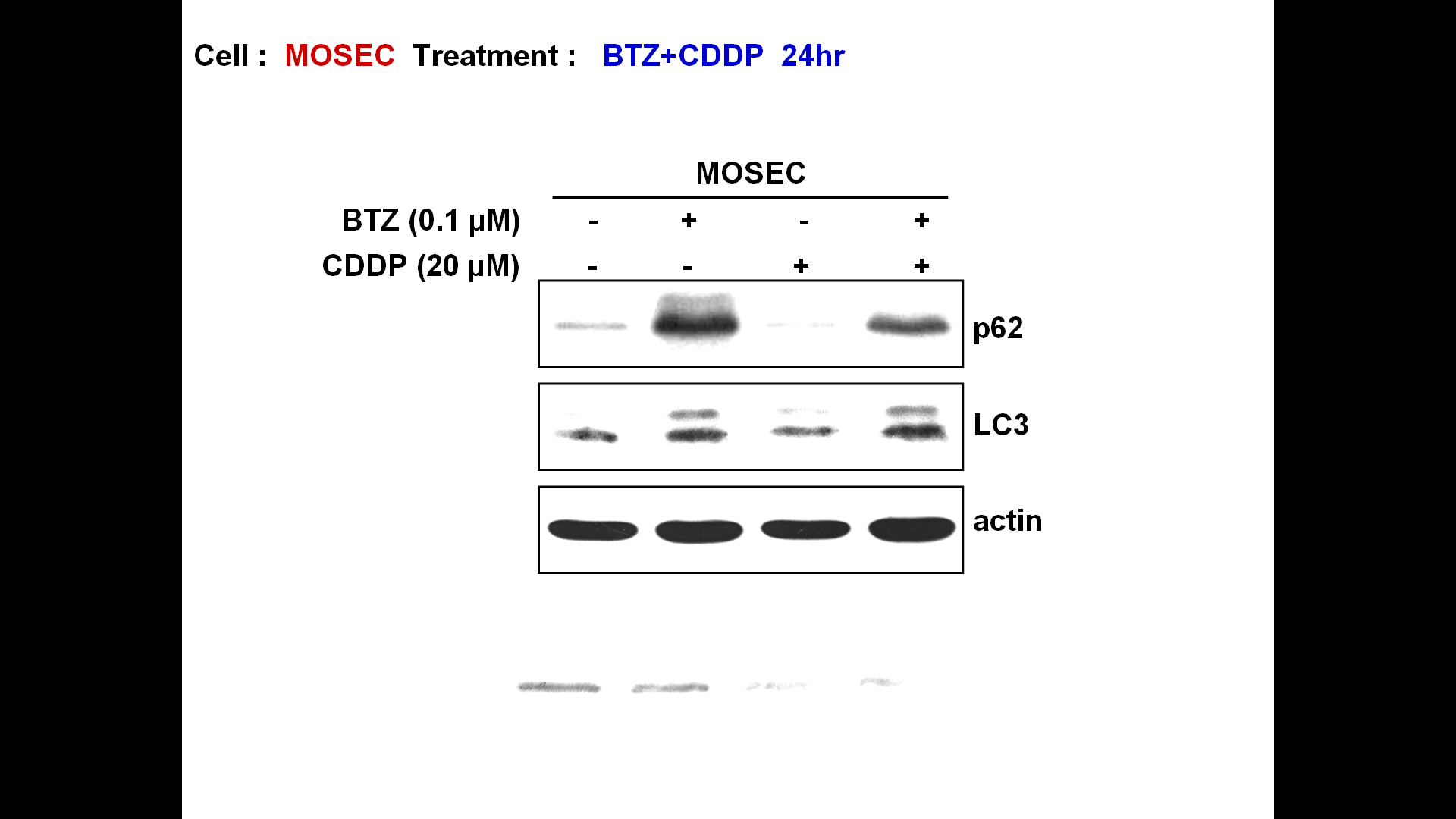
**
